# Early alterations in the MCH system link aberrant neuronal activity and sleep disturbances in a mouse model of Alzheimer’s disease

**DOI:** 10.1038/s41593-023-01325-4

**Published:** 2023-05-15

**Authors:** Sara Calafate, Gökhan Özturan, Nicola Thrupp, Jeroen Vanderlinden, Luísa Santa-Marinha, Rafaela Morais-Ribeiro, Antonella Ruggiero, Ivan Bozic, Thomas Rusterholz, Blanca Lorente-Echeverría, Marcelo Dias, Wei-Ting Chen, Mark Fiers, Ashley Lu, Ine Vlaeminck, Eline Creemers, Katleen Craessaerts, Joris Vandenbempt, Luuk van Boekholdt, Suresh Poovathingal, Kristofer Davie, Dietmar Rudolf Thal, Keimpe Wierda, Tiago Gil Oliveira, Inna Slutsky, Antoine Adamantidis, Bart De Strooper, Joris de Wit

**Affiliations:** 1 VIB Center for Brain & Disease Research, Leuven, Belgium; 2 KU Leuven, Department of Neurosciences, Leuven Brain Institute, Leuven, Belgium; 3 Life and Health Sciences Research Institute (ICVS), School of Medicine, University of Minho, Braga, Portugal; 4 ICVS/3B’s - PT Government Associate Laboratory, Braga/Guimarães, Portugal; 5 Department of Physiology and Pharmacology, Sackler Faculty of Medicine, Tel Aviv University, Tel Aviv, Israel; 6 Zentrum für Experimentelle Neurologie, Department of Neurology, Inselspital University Hospital Bern, University of Bern, Bern, Switzerland; 7 Department of Biomedical Research, University of Bern, Bern, Switzerland; 8 KU Leuven, Department of Otorhinolaryngology, Leuven, Belgium; 9 Department of Imaging and Pathology, Laboratory of Neuropathology, and Leuven Brain Institute, KU-Leuven, O&N IV, Leuven, Belgium; 10 Department of Pathology, UZ Leuven, Leuven, Belgium; 11 Sagol School of Neuroscience, Tel Aviv University, Tel Aviv, Israel; 12 UK Dementia Research Institute (UK DRI@UCL) at University College London, London, UK

**Keywords:** Cellular neuroscience, REM sleep, Excitability, Alzheimer's disease

## Abstract

Early Alzheimer’s disease (AD) is associated with hippocampal hyperactivity and decreased sleep quality. Here we show that homeostatic mechanisms transiently counteract the increased excitatory drive to CA1 neurons in *App*^NL-G-F^ mice, but that this mechanism fails in older mice. Spatial transcriptomics analysis identifies *Pmch* as part of the adaptive response in *App*^NL-G-F^ mice. *Pmch* encodes melanin-concentrating hormone (MCH), which is produced in sleep–active lateral hypothalamic neurons that project to CA1 and modulate memory. We show that MCH downregulates synaptic transmission, modulates firing rate homeostasis in hippocampal neurons and reverses the increased excitatory drive to CA1 neurons in *App*^NL-G-F^ mice. *App*^NL-G-F^ mice spend less time in rapid eye movement (REM) sleep. *App*^NL-G-F^ mice and individuals with AD show progressive changes in morphology of CA1-projecting MCH axons. Our findings identify the MCH system as vulnerable in early AD and suggest that impaired MCH-system function contributes to aberrant excitatory drive and sleep defects, which can compromise hippocampus-dependent functions.

## Abstract (web summary)

Increased neuronal activity and reduced sleep quality emerge in the early stages of Alzheimer’s disease (AD). Calafate et al. show that the sleep–active hypothalamic MCH system is involved in neuronal homeostasis but fails in the early stages of AD.

## Main

The prodromal phase is a long-lasting period of AD during which individuals remain cognitively stable despite the continuous accumulation of amyloid-β (Aβ) that correlates with neuronal hyperactivity^1–3^. In mouse models of Aβ accumulation, neuronal hyperactivity is prominent during low arousal states such as sleep and anesthesia^4^. Sleep disturbances with emergent silent epileptic-like discharges occur in individuals with AD during the prodromal phase^5–8^. The cognitive stability suggests that during this phase, homeostatic plasticity mechanisms are recruited to counteract deviations of neuronal activity from a physiological window^9,10^, thereby preventing earlier onset of cognitive decline. Homeostatic plasticity mechanisms remain poorly understood, but they are prominent during sleep and drive a net decrease in excitatory synaptic strength and neuronal firing rates^11–17^. Here we show that in *App*^NL-G-F^ mice homeostatic plasticity mechanisms are initially recruited in the CA1 region but are eventually insufficient to maintain excitatory drive at control levels. Using spatial transcriptomics, we identify MCH as a modulator of hippocampal synaptic transmission and firing rate homeostasis in *App*^NL-G-F^ mice. MCH-expressing neurons, which are located in the lateral hypothalamic area (LHA) and project to CA1, are active during sleep^18,19^ and modulate hippocampus-dependent memory^20^. We find that *App*^NL-G-F^ mice have a reduced fraction of active MCH neurons and impaired sleep–wake architecture. We show that MCH peptide is sufficient to reverse the increased excitatory drive in the CA1 region of *App*^NL-G-F^ mice. In both *App*^NL-G-F^ mice and brain samples from individuals with AD, we observe progressive defects in MCH axons. Together, our findings identify MCH as a vulnerable system in early AD and suggest a model in which impaired MCH-dependent synaptic function in CA1 and perturbed sleep–wake architecture synergistically compromise neuronal homeostasis.

## Results

### Dynamic changes in excitatory drive to *App*^NL-G-F^ CA1 neurons

**Fig. 1:**
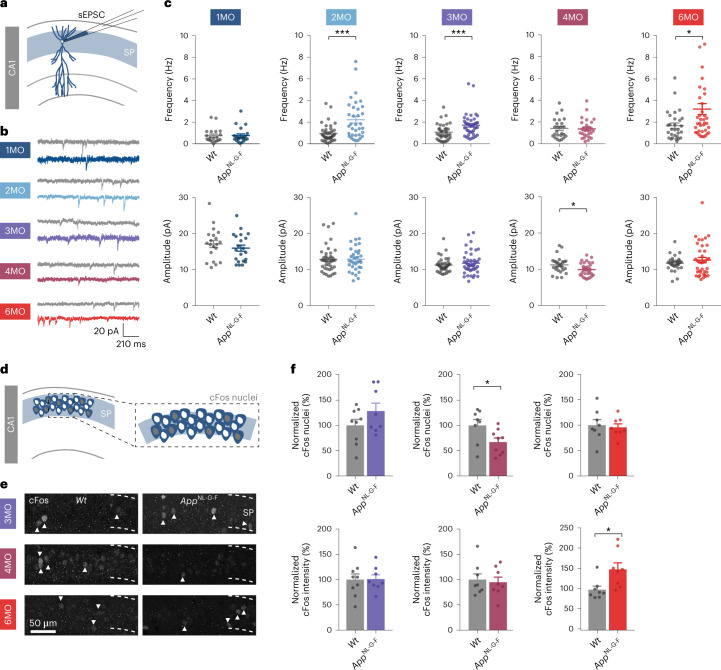
Homeostatic plasticity response counteracts increased excitatory drive to CA1 pyramidal neurons in *App*^NL-G-F^ mice. **a**–**c**, Whole-cell voltage clamp recordings of sEPSCs in CA1 pyramidal neurons in acute hippocampal slices from *Wt* and *App*^NL-G-F^ mice at different months (MO). Schematic (**a**) and representative traces (**b**) of analyzed frequency and amplitude of sEPSCs (**c**). Number of neurons from at least three mice per genotype: 1MO - *Wt* *n* = 20, *App*^NL-G-F^ *n* = 21; 2MO - *Wt* *n* = 40, *App*^NL-G-F^ *n* = 34 (*P* = 0.003); 3MO *- Wt* *n* = 36, *App*^NL-G-F^ *n* = 44 (*P* = 0.0008); 4MO *- Wt* *n* = 24, *App*^NL-G-F^ *n* = 27 (*P* = 0.0343), 6MO *- Wt* *n* = 29, *App*^NL-G-F^ *n* = 38 (*P* = 0.0168). Two-tailed unpaired *t*-test or Mann–Whitney test was used, depending on normality. Individual data points shown with bars represent the mean ± s.e.m. (*P < 0.05, ***P < 0.001). **d**–**f**, cFos immunostaining of CA1 neurons from *Wt* and *App*^NL-G-F^ mice at 3, 4 and 6 MO. Schematic (**d**) and representative images (**e**) of cFos in CA1 neurons at each MO. **f**, Top graphs show quantification of normalized number of cFos-positive CA1 neurons. Bottom graphs show the normalized intensity of the cFos signal in positive neurons. Number of mice: 3MO - *Wt* *n* = 9, *App*^NL-G-F^ *n* = 8; 4MO - *Wt* *n* = 8, *App*^NL-G-F^ *n* = 9 (*P* = 0.0335); 6MO - *Wt* *n* = 8, *App*^NL-G-F^ *n* = 8 (*P* = 0.0148). Two-tailed unpaired *t*-test or Mann–Whitney test was used, depending on normality. Individual data points are shown and bars represent the mean ± s.e.m. (*P < 0.05).

**Extended Data Fig. 1:**
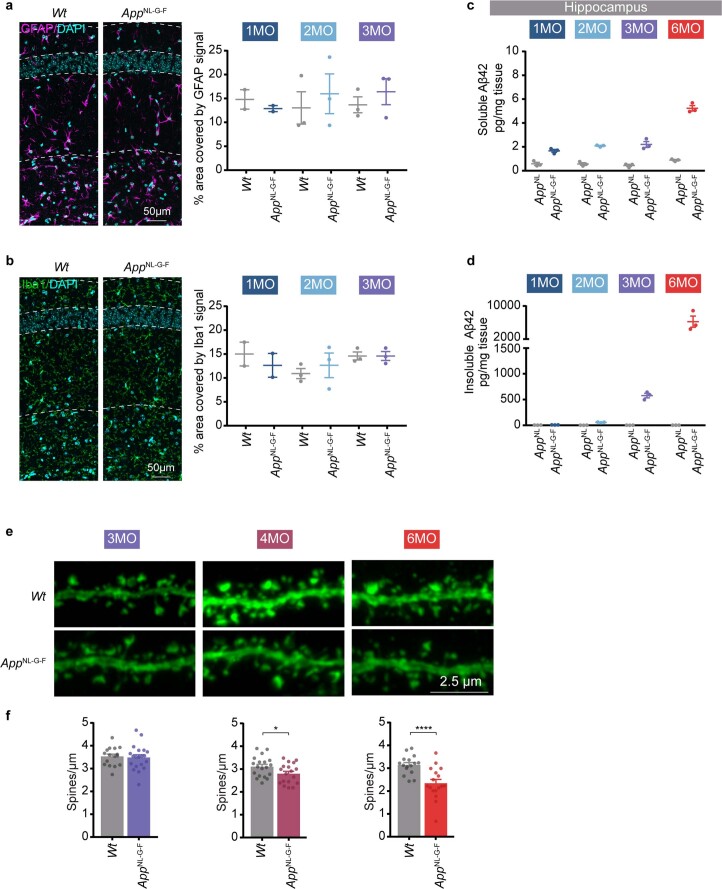
Early morphological alterations in the *App*^NL-G-F^ mice. **a,b**, Representative image and area covered by signal on CA1 hippocampal sections from *Wt* and *App*^NL-G-F^ mice at 1, 2 and 3 months (MO) immunostained for **(a)** astrocyte-marker GFAP (magenta) and **(b)** marker of microglia activation Iba1 (green). Nuclei are labeled with DAPI (cyan). n = 3 mice per time point and genotype. Individual data points shown with bars representing mean ± SEM. **c,d**, Quantification of **(c)** soluble and **(d)** insoluble Aβ_42_ using meso ELISA on *App*^NL^ and *App*^NL-G-F^ mice hippocampal lysates at 1, 2, 3 and 6 MO. Number of mice: n = 3 mice per time point and genotype. *App*^NL^ mouse was used as control as it contains the human *App* gene but with only one mutation and does not develop Aβ plaques compared to *App*^NL-G-F^. Individual data points shown with bars representing mean ± SEM. **e,f**, Spine analysis of CA1 pyramidal neuron proximal apical dendrites labelled with GFP from *Wt* and *App*^NL-G-F^ at different months (MO). **(e)** Representative images and **(f)** quantification of spine number per dendrite length. Number of dendrites from 3 mice per time point and genotype: 3MO *- Wt* n = 16, *App*^NL-G-F^ n = 20; 4MO - *Wt* n = 19, *App*^NL-G-F^ n = 18 (p = 0.0395), 6MO - *Wt* n = 16, *App*^NL-G-F^ n = 17 (p = 0.0005). Two-tailed unpaired *t*-test. Individual data points shown with bars representing mean ± SEM. (*p < 0.05, ****p < 0.0001).

An increase in cortical and hippocampal neuronal activity has been observed in different mouse models that accumulate Aβ^1,21,22^. We set out to investigate the onset and progression of altered activity in the CA1 region of the hippocampus in *App*^NL-G-F^ mice. We found an increased frequency of spontaneous excitatory postsynaptic currents (sEPSCs) in CA1 pyramidal neurons in acute *App*^NL-G-F^ mouse hippocampal slices that started as early as 2 months of age and persisted at 3 months (Fig. 1a–c), before deposition of Aβ and associated gliosis (Extended Data Fig. 1a–d). While Aβ accumulation was gradual, the increased excitatory drive in CA1 neurons strikingly fluctuated over time. Indeed, at 4 months, the frequency of sEPSCs was similar between genotypes, while sEPSC amplitude was transiently decreased in *App*^NL-G-F^ mice (Fig. 1a–c). At 6 months of age, sEPSC frequency in *App*^NL-G-F^ mice strongly increased again (Fig. 1a–c). The transient normalization of sEPSC frequency and decrease in sEPSC amplitude at 4 months suggest that homeostatic mechanisms were recruited at this stage to maintain CA1 neuronal excitatory synaptic transmission at control levels^9,23,24^.

**Extended Data Fig. 2:**
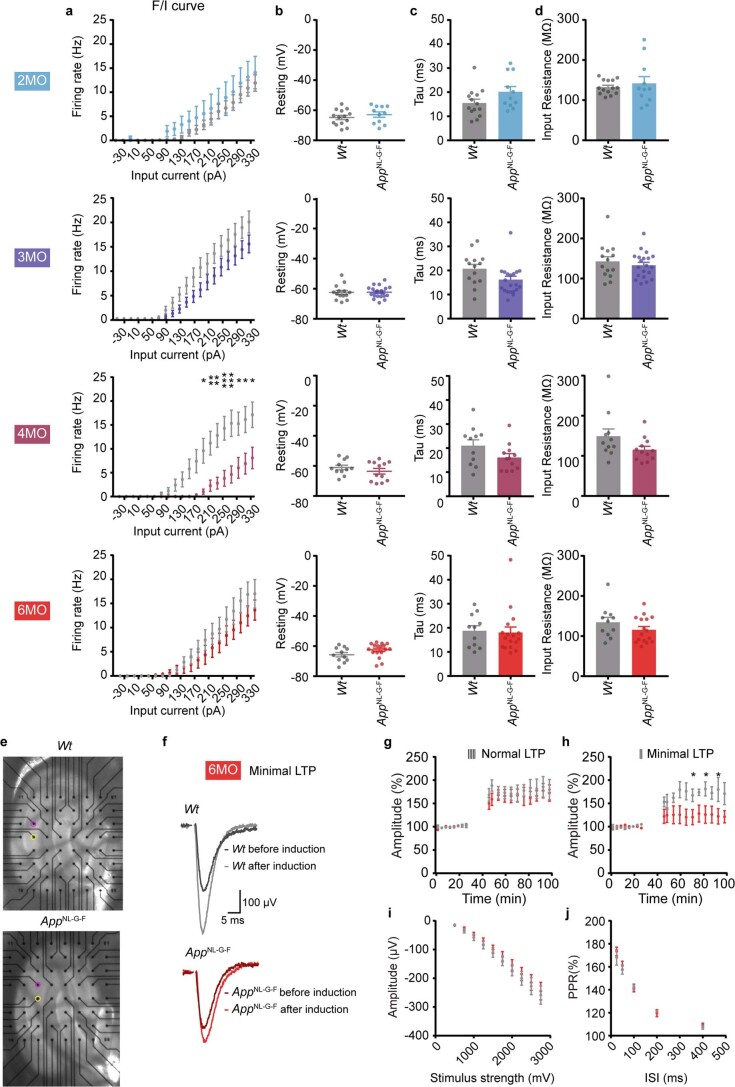
Early functional alterations in the *App*^NL-G-F^ mice. **a,b,c,d** Whole-cell current clamp recordings of intrinsic properties from CA1 pyramidal neurons from *Wt* and *App*^NL-G-F^ at different months (MO). **(a)** Intrinsic excitability, firing rate in function of somatic current injection, **(b)** resting membrane potential, **(c)** Tau membrane constant and **(d)** input resistance. Number of neurons from 3 or more mice, 3MO *- Wt* n = 14, *App*^NL-G-F^ n = 20; 4MO - *Wt* n = 11, *App*^NL-G-F^ n = 12, 6MO - *Wt* n = 11, *App*^NL-G-F^ n = 17. Two-tailed unpaired *t*-test. (*p < 0.05). **(a)** Two-way ANOVA, with Holm-Sidak *post-hoc* multiple comparisons test (190 pA p = 0.0157, 210 pA p = 0.0098, 230 pA p = 0.0018, 250 and 270 pA p = 0.0008, 290 pA p = 0.0171, 310 pA p = 0.0212, 330 pA p = 0.0303)). **(b,c,d)** Two-tailed unpaired *t*-test. Individual data points shown with bars representing mean ± SEM. (*p < 0.05, **p < 0.01, ***p < 0.001). **e**, Images of *Wt* (left) and *App*^NL-G-F^ (right) acute hippocampal slices on the multielectrode array (MEA2100, Multichannel Systems) used for field excitatory postsynaptic potential (fEPSP) recordings. Stimulation and recording electrodes are indicated with purple and yellow circles, respectively. **f**, Example traces of fEPSP responses in *Wt* and *App*^NL-G-F^ before (dark traces) and 55 minutes after minimal LTP induction (light traces). **g**, LTP induced in CA1 region by Schaffer collateral (SC) pathway stimulation using 3 theta burst stimulations. **h**, LTP induced in CA1 region using minimized theta burst stimulation paradigm (65.05 min p = 0.048, 81.55 min p = 0.049, 92.55 min p = 0.0366). **i**, Input-output (IO) relationship for *Wt* and *App*^NL-G-F^ slices. **j**, Paired-pulse ratio (PPR) stimulations of SC pathway. Stimulation intensities used for LTP and paired pulse ratio recordings were calculated from CA1 region using SC stimulation in *Wt* and *App*^NL-G-F^ slices (inter stimulus intervals (ISI): 25, 50, 100, 200 and 400 ms). Number of mice: Normal LTP –*Wt* n = 5, *App*^NL-G-F^ n = 5; Minimal LTP - *Wt* n = 4, *App*^NL-G-F^ n = 4. Two-way ANOVA, with Holm-Sidak *post-hoc* multiple comparisons test. Individual data points shown with bars representing mean ± SEM. (*p < 0.05).

We next analyzed cell-intrinsic properties of CA1 neurons and found a markedly reduced intrinsic excitability specifically at 4 months in *App*^NL-G-F^ mice (Extended Data Fig. 2a–d). To independently assess the activity of neurons in the CA1 pyramidal layer over time, we performed immunohistochemistry for the activity-regulated immediate early gene cFos. We observed a decrease in the percentage of cFos-positive (active) CA1 neurons selectively at 4 months in *App*^NL-G-F^ mice (Fig. 1d–f). These observations support the view that homeostatic mechanisms are acting on CA1 pyramidal neurons at this stage in response to an increased excitatory drive in *App*^NL-G-F^ mice.

Interestingly, the cFos signal intensity increased at 6 months (Fig. 1d–f), in parallel with a rise in sEPSC frequency (Fig. 1a–c). Moreover, we observed a progressive decrease in spine density in proximal apical dendrites from *App*^NL-G-F^ CA1 pyramidal neurons that started at 4 months and further decreased at 6 months (Extended Data Fig. 1e,f), as well as an increased threshold for long-term potentiation (LTP) at Schaffer collateral–CA1 synapses at 6 months (Extended Data Fig. 2e–j)^24,25^. Together, these results indicate that homeostatic responses are initially recruited between 3 and 4 months to counteract an increase in excitatory synaptic transmission in CA1 neurons, but that these are not sufficient to stabilize synaptic transmission at control levels at 6 months in response to continuous Aβ accumulation.

### Synaptic plasticity signature in *App*^NL-G-F^ CA1 pyramidal layer

**Fig. 2:**
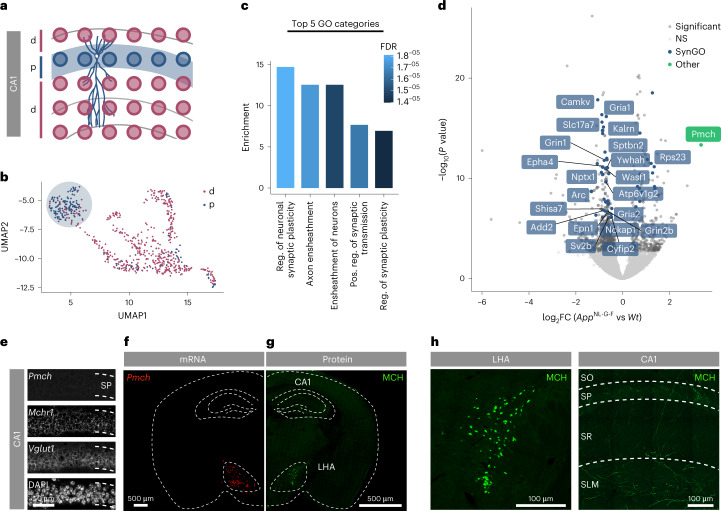
Spatial transcriptomics reveals *Pmch* as a key player in plasticity response. **a**,**b**, Spatial transcriptomics performed on mouse sections of *Wt* and *App*^NL-G-F^ brains at 3.5 MO. **a**, TDs from CA1 pyramidal layer (p) (**b**) cluster away from dendritic (d) TDs in an unbiased cluster analysis. Number of TDs in p: *Wt* *n* = 33, *App*^NL-G-F^ *n* = 42. **c**, Results of GO enrichment analysis on the top 200 DE genes (based on *P* value). GO categories were sorted by *P* value and the top 8 GO categories were ordered by normalized enrichment score (Fisher’s exact test). The five most enriched GO categories are shown in the bar plot. Coloring represents false discovery rate (FDR). See additional information in Supplementary Table 2. **d**, Volcano plot showing average gene expression differences between *App*^NL-G-F^ and *Wt* TDs. Significant genes (EdgeR’s quasi-likelihood *F* test with Benjamini–Hochberg correction < 0.05), are shown in dark gray. Significant genes annotated in SynGO are highlighted in blue, other significant genes of interest are shown in green. The 21 genes annotated in SynGO and present in at least one homeostatic plasticity dataset are labeled (Supplementary Table 3). NS, not significant. **e**, CA1 pyramidal layer crops showing *Pmch*, *Mchr1* and *Vglut1* (*Slc17a7*) mRNA expression using RNAscope. DAPI was used to label nuclei. *Wt* *n* = 2 independent experiments. **f**,**g**, Whole-brain coronal section showing expression of *Pmch* (**f**) and MCH (**g**). *Wt* *n* = 3 independent experiments. **h**, Crops of LHA and CA1 regions showing MCH-positive cell bodies in the LHA and axons projecting to CA1 region. *Wt* *n* = 3 independent experiments. FC, fold change.

**Extended Data Fig. 3:**
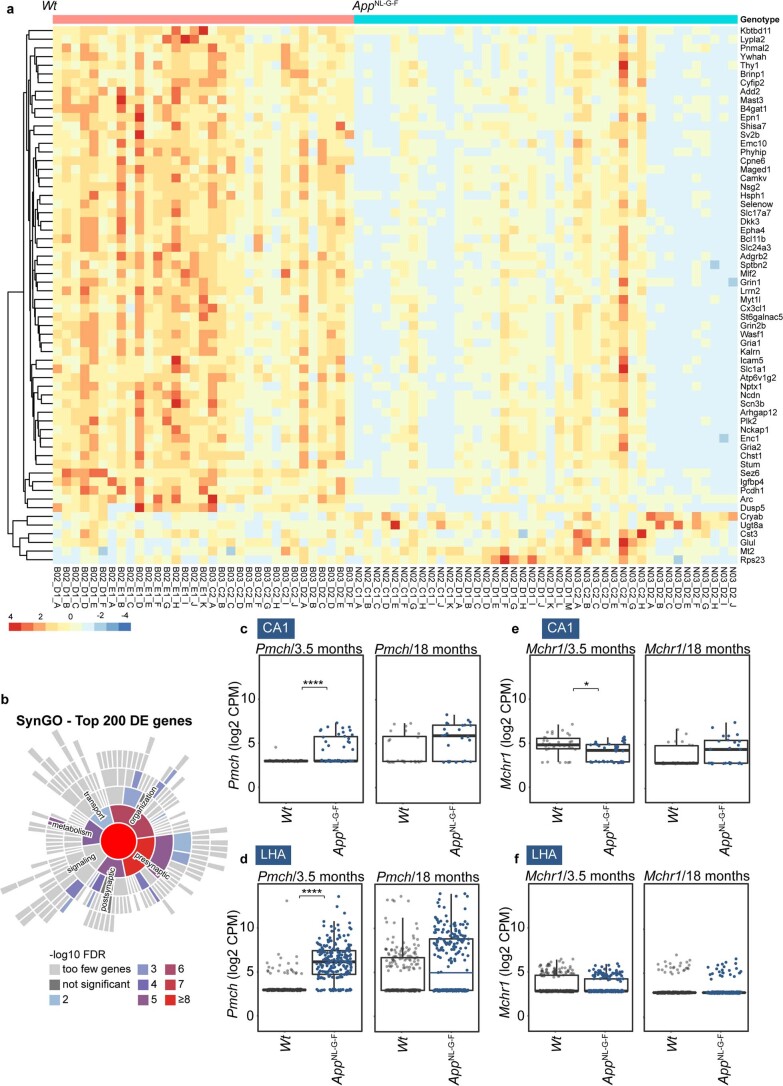
Transcriptional alterations in the *App*^NL-G-F^ mice. **a**, Heatmap showing scaled expression of DE genes (in Spatial Transcriptomics) that appear in at least one of four homeostatic plasticity datasets^27–30^. Columns represent tissue domains (TD) taken from the SP layer of CA1 region, from multiple tissue slides of 2 *App*^NL-G-F^ mice and 2 *Wt* mice at 3.5 months (mouse genotypes are shown as colored bars across the top). Mouse information metadata is available in Supplementary Table 4. **b**, Sunburst visualization of Synaptic Gene Ontology (SynGO) enriched ontology terms on the top 200 DE genes (based on p-value), using a one-sided Fisher’s Exact Test, colored by -log10 FDR (False Discovery Rate). **c,d,e,f**, Spatial Transcriptomics performed on coronal mouse sections of *Wt* and *App*^NL-G-F^ brains at 3.5 and 18 months. Tissue domains (TD) from CA1 pyramidal layer and LHA were selected and *Pmch* and *Mchr1* mRNA levels analyzed. Number of TDs in CA1 pyramidal layer: 3.5 months - *Wt* n = 33, *App*^NL-G-F^ n = 42 (*Pmch* p = 0.0001, *Mchr1* p = 0.0318); 18 months - *Wt* n = 25, *App*^NL-G-F^ n = 25; Number of TDs in LHA: 3.5 months - *Wt* n = 418, *App*^NL-G-F^ n = 383 (*Pmch* p = 0.0001); 18 months - *Wt* n = 280, *App*^NL-G-F^ n = 365; EdgeR’s quasi-likelihood F-test (*p < 0.05, ****p < 0.0001). Boxplots show medians, interquartile ranges and minimum/maximum values (up to 1.5 x interquartile range) of log2 normalised expression per TD.

To determine whether we could detect evidence of a homeostatic response in the CA1 pyramidal layer in *App*^NL-G-F^ mice at the molecular level, we analyzed our spatial transcriptomics dataset at 3.5 months of age^26^. We retrieved small tissue domains (TDs) that cover the pyramidal layer containing cell bodies of CA1 neurons from wild-type (*Wt*) and *App*^NL-G-F^ mice (Fig. 2a,b) and quantified differentially expressed (DE) transcripts between genotypes (Supplementary Table 1). Gene Ontology (GO) analysis of the top 200 DE genes (sorted on *P* value) identified ‘regulation of neuronal synaptic plasticity’ as the top GO category, with three of five top enriched GO categories related to synaptic processes (Fig. 2c and Supplementary Table 2). We then cross-referenced the top 200 DE genes with four transcriptomics and proteomics datasets of classical models for homeostatic synaptic plasticity^27–30^ (Extended Data Fig. 3a) and found that 62 DE genes were modulated in at least one of these datasets. From these, 21 genes also have an annotated synaptic function in the SynGO database (Extended Data Fig. 3b, Supplementary Table 3 and Fig. 2d). These include genes such as *Arc*, *Nptx1* and *Epha4* that are modulated upon changes in neuronal activity^27–31^, as well as *Gria1* and *Gria2* encoding AMPA receptor (AMPAR) subunits, all of which are downregulated in the CA1 pyramidal layer of *App*^NL-G-F^ mice (Fig. 2d). Thus, consistent with our electrophysiological and immunohistochemical observations, spatial transcriptomics analysis revealed a molecular signature of a homeostatic synaptic plasticity response in the *App*^NL-G-F^ CA1 pyramidal layer at 3.5 months of age.

### Upregulation of *Pmch* in the *App*^NL-G-F^ CA1 pyramidal layer

**Extended Data Fig. 4:**
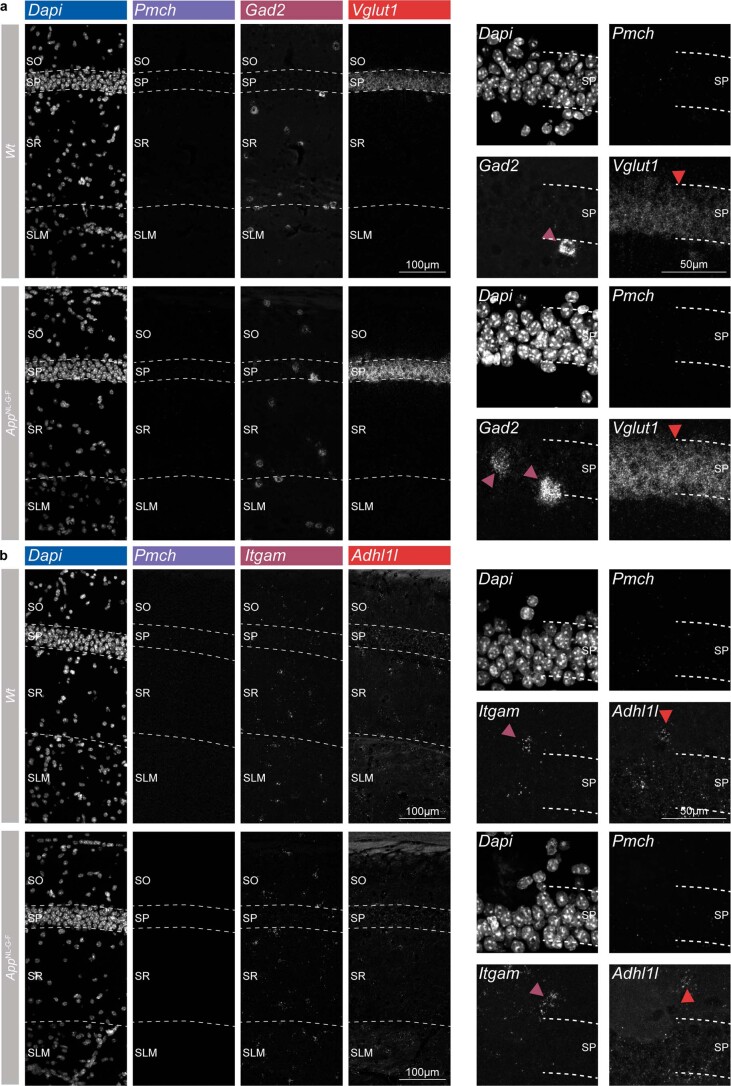
*Pmch* is not expressed in CA1 cell-bodies. **a**, RNAscope on 10μm *Wt* and *App*^NL-G-F^ in mouse brain coronal sections at 3.5 months showing *Pmch, Gad2*, and *Vglut1* *(Slc17a7)* expression in CA1 region. **b**, *Pmch, Itgam* and *Adhl1l* expression in CA1 region. A higher magnification image is shown on the right side for each of the transcripts.

**Extended Data Fig. 5:**
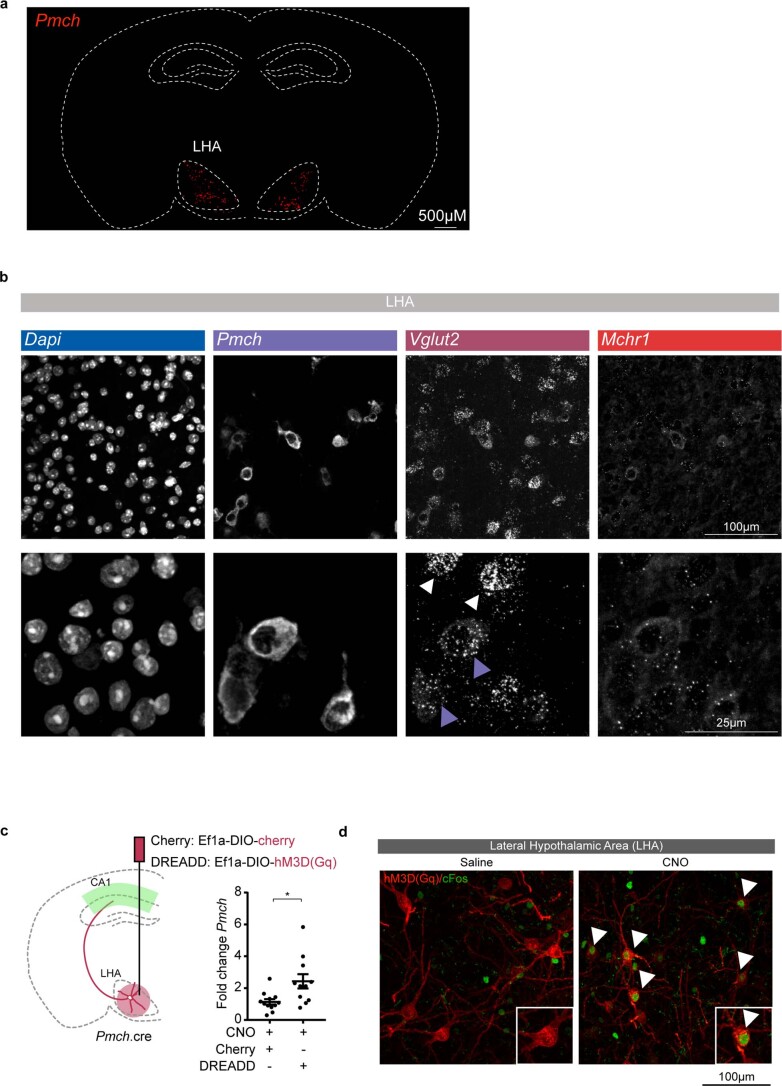
*Pmch* is expressed in LHA cell-bodies. **a,b**, RNAscope on 10μm *Wt* mouse brain coronal section showing **(a)** *Pmch* expression in whole section and **(b)** *Pmch*, *Vglut2 (Slc17a6)* and *Mchr1* expression in the lateral hypothalamic area (LHA) (top row) and highlighted in crops (bottom row). White arrowheads indicate *Vglut2* positive cells that do not express *Pmch*. Purple arrowheads indicate *Vglut2-* and *Pmch*-positive cells. **c**, RT-qPCR on hippocampal extracts from *Pmch*.cre animals expressing Cherry or hM3Dq(Gq) (DREADD-Cherry) in MCH-neurons in the LHA. CNO was i.p. injected at the beginning of the light-phase for 4 hours (3 mg/kg). Number of mice: Cherry n = 12, DREADD n = 11 (p = 0.0115). Two-tailed unpaired t-test. Individual data points shown with bars representing mean ± SEM. (*p < 0.05). **d**, Representative images from *Pmch*.cre animal expressing hM3Dq(Gq) in MCH-neurons in the LHA. Saline or CNO were i.p. injected at the beginning of the light-phase for 4 hours (3 mg/kg), to show that CNO injection induces cFos expression in hM3Dq(Gq)-expressing neurons. Arrowheads indicate active MCH-neurons that express hM3Dq(Gq) (red) and cFos (green). These animals belong to a different cohort from the animals used in Extended Data Fig. 5c. Number of mice: Saline n = 1, CNO n = 1.

An advantage of the spatial transcriptomics approach is that it covers cellular niches in the brain, yielding the transcriptional profiles of CA1 pyramidal neurons but also of the surrounding environment^26^. Spatial transcriptomics analysis revealed that the most upregulated gene in the CA1 pyramidal region of *App*^NL-G-F^ mice at 3.5 months is *Pmch*, which belongs to the top GO category (regulation of neuronal synaptic plasticity; Fig. 2c,d and Supplementary Table 2). *Pmch* encodes the prepro-melanin-concentrating hormone peptide, which is further processed into several peptides including MCH^32,33^. While no precise hippocampal synaptic function has been attributed to this peptide, the involvement of its functional MCH receptor 1 (MCHR1) in plasticity has been suggested^34,35^. Moreover, injection of MCH in the hippocampus promotes memory^36,37^ and MCH-positive hypothalamic neurons play a role in novelty detection^38,39^ and memory consolidation during sleep^20^. *Pmch* mRNA is primarily expressed in neurons located in the LHA^33^, confirmed by single-molecule fluorescence in situ hybridization and available single-cell sequencing datasets (Fig. 2e,f and Extended Data Figs. 4 and 5; https://celltypes.brain-map.org/rnaseq/)^40^. Neither excitatory and inhibitory neurons nor glial cells in *Wt* or *App*^NL-G-F^ CA1 expressed *Pmch* (Extended Data Fig. 4a,b). MCH neurons in the LHA (Fig. 2g) broadly project their MCH-rich axonal terminals, including to the dorsal CA1 region, as shown by MCH immunohistochemistry in sections of adult *Wt* mice (Fig. 2h), consistent with previous studies^33^. Therefore, elevated *Pmch* expression detected in the CA1 spatial transcriptomics analysis points to an increase in *Pmch* mRNA pools in MCH-positive axons. CA1 pyramidal neurons do express *Mchr1* (Fig. 2e). Spatial transcriptomics analysis of *Pmch* and *Mchr1* levels in the CA1 region showed that *Pmch* is upregulated at 3.5 months but not at 18 months, whereas *Mchr1* is downregulated at 3.5 months but not at 18 months in *App*^NL-G-F^ mice (Extended Data Fig. 3c–f). Spatial transcriptomics analysis further revealed that *Pmch* is also upregulated in the LHA of *App*^NL-G-F^ mice at 3.5 months (Extended Data Fig. 3c–f).

To test whether manipulating the activity of MCH neurons in the LHA affects *Pmch* mRNA levels in the hippocampus, we expressed hM3Dq(Gq) designer receptors exclusively activated by designer drugs (DREADDs) in the LHA of *Pmch*-cre mice. Acute activation of MCH neurons by clozapine *N*-oxide (CNO; 3 mg per kg body weight, intraperitoneal injection) resulted in an increase in *Pmch* mRNA levels in the hippocampus (Extended Data Fig. 5c,d). Altogether, these results suggest that *Pmch* can be dynamically regulated in MCH axons located in the CA1 region.

### Melanin-concentrating hormone reduces synaptic strength and modulates mean firing rate

**Fig. 3:**
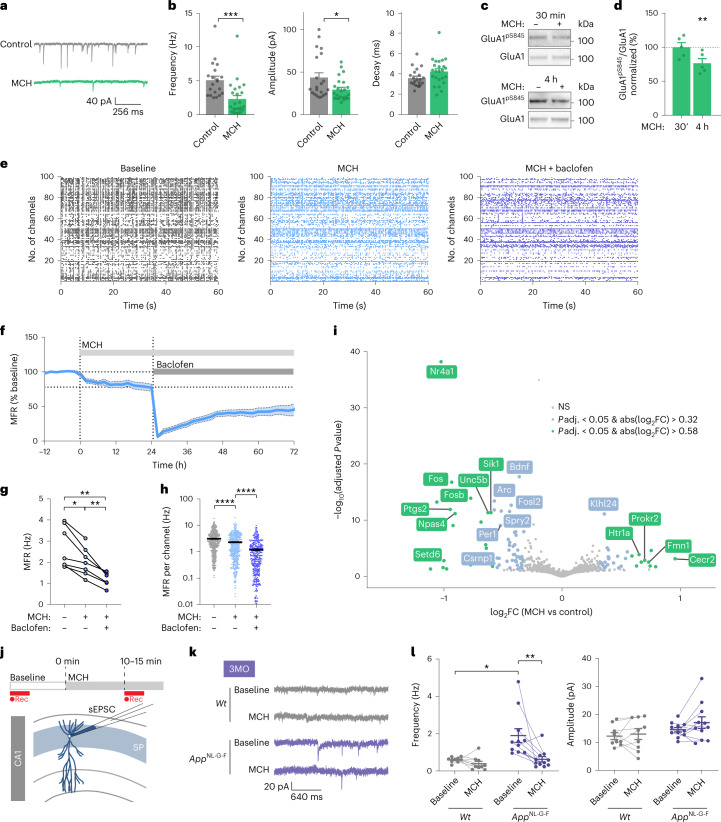
Melanin-concentrating hormone decreases synaptic strength and modulates firing rate homeostasis. **a**,**b**, Whole-cell voltage clamp recordings of sEPSCs in hippocampal cultured neurons treated with vehicle or 1 μM MCH peptide for 4 h. Representative raw traces (**a**) and graphs (**b**) of sEPSC frequency (*P* = 0.0001), amplitude (*P* = 0.0255) and decay time. Number of independent cultures, *n* = 4; number of neurons, control *n* = 20, 1 μM MCH *n* = 24. Two-tailed unpaired *t*-test or Mann–Whitney test, depending on normality. Individual data points shown; bars represent the mean ± s.e.m. (*P < 0.05, ***P < 0.001). **c**,**d**, Hippocampal cultures treated with vehicle or 1 μM MCH for 30 min or 4 h (**c**) analyzed for phosphorylated GluA1 on serine 845 (GluA1^pSer845^) and total GluA1 levels. **d**, GluA1^pSer845^/GluA1 ratio normalized to vehicle (*P* = 0.0079). Number of independent cultures for all conditions, *n* = 5; data points represent the average of three replicas per each independent culture. Two-tailed unpaired *t*-test. Individual data points shown; bars represent the mean ± s.e.m. (**P < 0.01). **e**, Raster plots from a representative MEA experiment in hippocampal neurons showing activity of the same 99 channels in baseline, 24 h of 1 µM MCH and 2 d after application of 10 µM baclofen (MCH + baclofen). **f**, Time course of MFR after MCH (1 µM, ~20% steady-state reduction) and impaired renormalization of MFR after baclofen (10 µM) to the new set point. **g**, Summary of MFRs following 24 h of MCH and MCH + baclofen for 2 d. Number of independent cultures: baseline, *n* = 7; MCH, *n* = 7; baclofen, *n* = 6. (Baseline versus MCH *P* = 0.0151; MCH versus baclofen *P* = 0.0048, baseline versus baclofen *P* = 0.0099). Mixed-effect model analysis with Tukey’s post hoc test. (*P < 0.05, **P < 0.01). **h**, Changes in MFR per channel after MCH (MCH, 2.30 ± 0.11 Hz) and MCH + baclofen for 2 d (MCH + baclofen, 1.2 ± 0.08 Hz) compared to baseline (baseline, 3.12 ± 0.13 Hz). Number of independent cultures: baseline, *n* = 7; MCH, *n* = 7; baclofen, *n* = 6. (Baseline versus MCH *P* < 0.0001; MCH versus baclofen *P* < 0.0001, baseline versus baclofen *P* < 0.0001). Individual data points shown with bars representing the mean ± s.e.m. Mixed-effect model analysis with Tukey’s post hoc test (****P < 0.0001). **i**, Volcano plot showing gene expression differences between hippocampal cultures treated with vehicle or 1 μM MCH for 4 h. Number of independent cultures: *n* = 2, 2 replicas per culture. (log_2_FC values were calculated using DESeq2. *P* values were calculated using the Wald test and adjusted for multiple testing using Benjamini–Hochberg correction). DE genes are available in Supplementary Table 5. **j**–**l**, Whole-cell voltage clamp recordings of sEPSC in CA1 pyramidal neurons in acute hippocampal slices before (baseline) and after incubation of 1 µM of MCH, from *Wt* and *App*^NL-G-F^ mice at 3 MO. Schematic (**j**) and representative traces (**k**) of sEPSC frequency (**l**; *Wt* baseline versus *App*^NL-G-F^ baseline *P* = 0.0239; *App*^NL-G-F^ baseline versus *App*^NL-G-F^ MCH *P* = 0.0032) and amplitude. Number of neurons: 3MO neurons from six or more mice *- Wt* *n* = 9, *App*^NL-G-F^ *n* = 11. Two-tailed unpaired *t*-test or Mann–Whitney test used depending on normality. Individual data points shown; bars represent the mean ± s.e.m. (*P < 0.05, **P < 0.01). Source data

**Extended Data Fig. 6:**
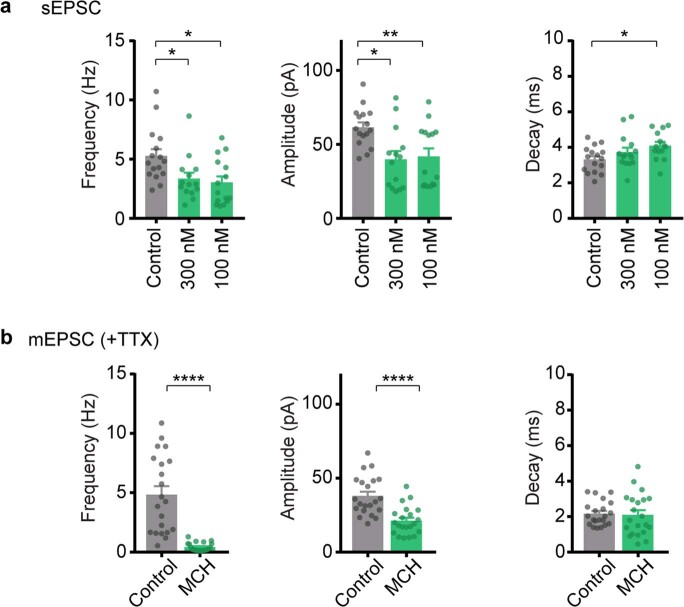
MCH decreases hippocampal synaptic strength. **a**, Whole-cell voltage clamp recordings of spontaneous excitatory postsynaptic currents (sEPSC) in hippocampal cultured neurons treated with control vehicle (H_2_O), 300 nM or 100 nM MCH peptide for 4 hours. (Frequency: 300 nM p = 0.0123, 100 nM p = 0.0455; Amplitude: 300 nM p = 0.0376; 100 nM p = 0.0137; Decay: 100 nM p = 0.04). Number of independent cultures: n = 3; Number of neurons: Control n = 16; 300 nM MCH n = 14; 100 nM MCH n = 13. Depending on normality, one-way ANOVA with Tukey’s post-hoc test or Kruskal-Wallis statistical test. Individual data points shown with bars representing mean ± SEM. (*p < 0.05, **p < 0.01). **b**, Whole-cell voltage clamp recordings of miniature excitatory post-synaptic currents (mEPSC) in the presence of tetrodotoxin (TTX, 1 μM) in hippocampal cultured neurons treated with control vehicle (H_2_O) or 1 μM MCH peptide for 4 hours. (Frequency: p = 0.0001; Amplitude: p = 0.0001). Number of independent cultures: n = 3; Number of neurons: Control n = 21; 1 μM MCH n = 21. Two-tailed unpaired *t*-test or Mann-Whitney test was used, depending on normality. Individual data points shown with bars representing mean ± SEM. (****p < 0.0001).

*Pmch* belongs to the most enriched GO category related to synaptic plasticity and was strongly upregulated in the *App*^NL-G-F^ LHA and CA1 regions at the stage when CA1 pyramidal neurons displayed a decrease in excitatory synaptic transmission, intrinsic excitability and cFos-positive neuron number. We thus reasoned that the MCH peptide might play a role in the observed homeostatic response, a previously unexplored question. To gain insight of the effect of MCH on hippocampal synaptic function, we first treated cultured *Wt* hippocampal neurons with different concentrations of MCH peptide (100 nM, 300 nM and 1 µM) (Fig. 3a,b and Extended Data Fig. 6a). MCH application strongly reduced excitatory synaptic transmission, as shown by a decrease in the frequency and amplitude of sEPSCs and miniature excitatory postsynaptic currents (mEPSCs; Fig. 3a,b and Extended Data Fig. 6b). The robust decrease in mEPSC amplitude indicates a reduction in postsynaptic AMPAR content following MCH treatment. Consistent with this, the levels of phosphorylated GluA1 on serine 845 (pS845), a phospho-epitope on the GluA1 AMPAR subunit that promotes surface targeting or retention, were decreased upon 4 h of MCH treatment (Fig. 3c,d). These results indicate that MCH reduces excitatory synaptic strength in hippocampal neurons.

Next, we treated cultured *Wt* hippocampal neurons grown on a multielectrode array (MEA) with MCH peptide and recorded neuronal activity over 24 h. MCH application caused a sustained decrease in mean firing rates (MFRs; Fig. 3e). To test whether MCH modulates homeostatic mechanisms, we used the GABA_B_ receptor agonist baclofen, previously shown to induce homeostatic mechanisms enabling recovery of MFR to a set-point level in response to inhibition^41^^,42^. Addition of 10 µM baclofen in the presence of MCH resulted in a transient reduction of MFR, followed by renormalization of the MFR to a new, lower set-point level (Fig. 3e–h). These results indicate that MCH peptide limits activity-dependent compensatory mechanisms underlying MFR renormalization.

To reveal the molecular correlates of MCH-induced responses, we performed bulk RNA sequencing (RNA-seq) on hippocampal neurons treated with MCH (Fig. 3i and Supplementary Table 5). MCH treatment consistently downregulated several genes involved in neuronal excitability and synaptic plasticity, including the immediate early genes *Nr4a1* (the most downregulated gene), *Fos*, *Fosb*, *Fosl2*, *Arc* and *Npas4* (refs. ^43–46^; Fig. 3i and Supplementary Table 5), and *Bdnf* and *Ptgs2* (refs. ^47,48^). Moreover, MCH application induced downregulation of *Spry2*, *Ptgs2* and *Sik1* and upregulation of *Prokr2* and *Klhl24—*changes that have previously been shown to protect neurons from excitotoxicity during kainate-mediated responses and epilepsy^49–53^ (Fig. 3i and Supplementary Table 5). Altogether, these observations suggest that MCH, released from MCH axons in the CA1 region, can protect CA1 pyramidal neurons from aberrant excitation through reduction of synaptic strength, MFR and expression of neuronal excitability-associated and synaptic plasticity-associated genes.

### Melanin-concentrating hormone reverses increased excitatory drive in *App*^NL-G-F^ CA1

To test whether MCH can reverse the increased excitatory synaptic drive of CA1 pyramidal neurons in *App*^NL-G-F^ mice, we recorded sEPSCs from CA1 pyramidal neurons in acute hippocampal slices from 3-month-old *Wt* and *App*^NL-G-F^ mice before and upon bath application of MCH peptide. We observed that acute MCH treatment reduced the elevated frequency of sEPSCs in *App*^NL-G-F^ CA1 pyramidal neurons to the baseline levels observed in *Wt* mice before MCH incubation, without affecting their amplitude (Fig. 3j–l). This result indicates that MCH can renormalize increased excitatory synaptic transmission in *App*^NL-G-F^ CA1 pyramidal neurons.

### Reduced fraction of active melanin-concentrating hormone neurons in *App*^NL-G-F^ mice

**Fig. 4:**
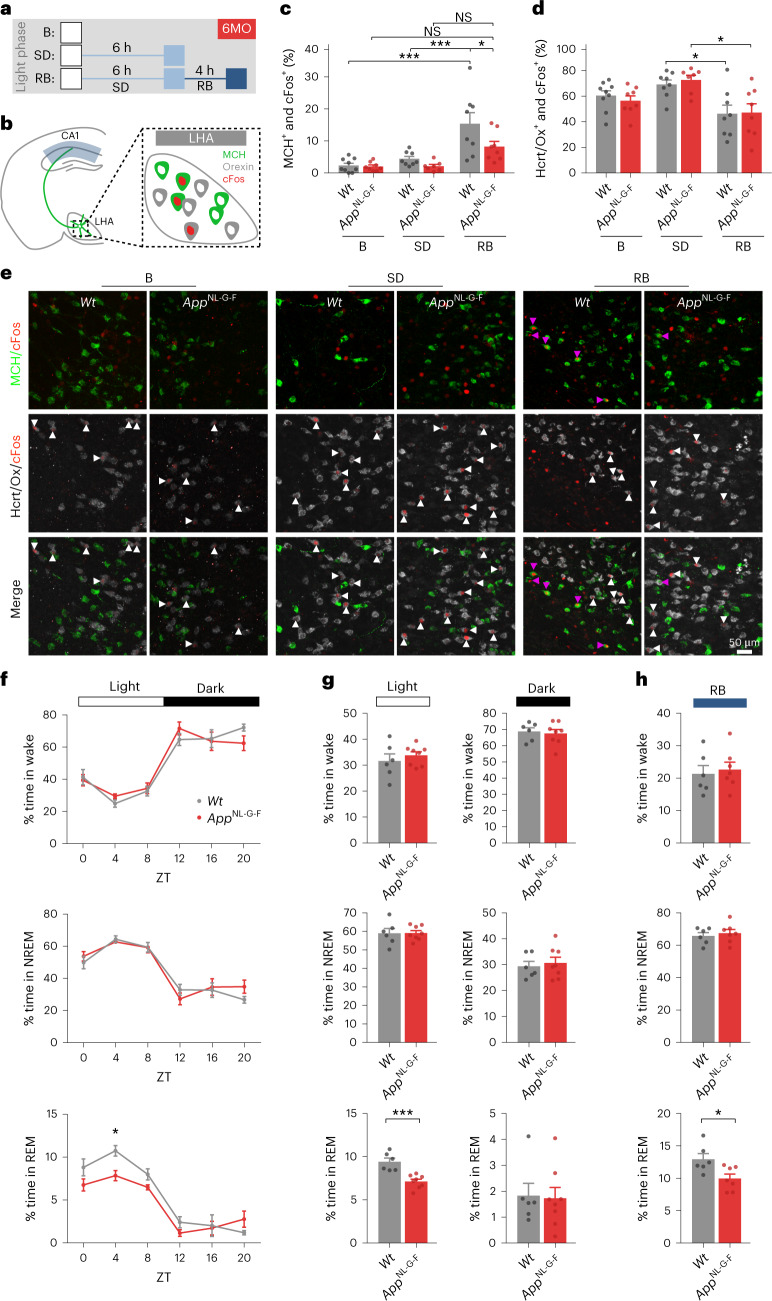
Reduced fraction of active MCH neurons and perturbed rapid eye movement sleep in *App*^NL-G-F^ mice. **a**, Schematic of experimental groups at 6 months: control (B), 6 h of SD, and 4 h of RB sleep following 6 h of SD. **b**, Location of analyzed neurons. **c**,**d**, Quantifications of LHA neurons from *Wt* and *App*^NL-G-F^ mice. **c**, MCH^+^ and cFos^+^ (*Wt* B versus *Wt* RB *P* = 0.0001, *Wt* SD versus *Wt* RB *P* = 0.0004, *Wt* RB versus *App*^NL-G-F^ RB *P* = 0.0448). **d**, Hcrt/Ox^+^ and cFos^+^ (*Wt* SD versus *Wt* RB *P* = 0.0354, *App*^NL-G-F^ SD versus *App*^NL-G-F^ RB *P* = 0.0126). Number of mice: 6 MO - B: *Wt* *n* = 9, *App*^NL-G-F^ *n* = 8; SD: *Wt* *n* = 8, *App*^NL-G-F^ *n* = 7, RB: *Wt* *n* = 8, *App*^NL-G-F^ *n* = 8. One-way analysis of variance (ANOVA) with Tukey’s post hoc test. Individual data points are shown; bars represent the mean ± s.e.m. (*P < 0.05, ***P < 0.001). **e**, Representative images of LHA show MCH (green) or Hcrt/Ox (gray) neurons positive for cFos (red). Magenta arrowheads denote MCH^+^ and cFos^+^; white arrows denote Hcrt/Ox^+^ and cFos^+^ neurons. **f**,**g**, Basal EEG/EMG recordings show the percentage of time spent in wake, NREM and REM states over Zeitgeber time (ZT; every 4 h; *P* = 0.0192; **f**) or during total light and dark phases (*P* = 0.0004; **g**). Number of mice: 6 MO - B: *Wt* *n* = 6, *App*^NL-G-F^ *n* = 8. Two-way ANOVA, with Holm–Sidak post hoc multiple-comparisons test. Individual data points are shown; bars represent the mean ± s.e.m. (*P < 0.05). **h**, EEG/EMG recordings during RB sleep show the percentage of time spent in wake, NREM or REM (*P* = 0.0214). Number of mice: 6 MO - RB: *Wt* *n* = 6, *App*^NL-G-F^ *n* = 7. Two-tailed unpaired *t*-test. Individual data points are shown; bars represent the mean ± s.e.m. (*P < 0.05).

Our findings show that MCH downregulates synaptic strength and MFR, modulates the homeostatic machinery in hippocampal neurons and reverses aberrant excitatory drive of *App*^NL-G-F^ CA1 pyramidal neurons. We next asked whether the activity of MCH neurons is altered in *App*^NL-G-F^ mice compared to *Wt* mice at 6 months of age, a time when CA1 excitatory drive stability is lost. MCH neurons are maximally active during rapid eye movement (REM) sleep^54^ and are intermingled with wake state-promoting hypocretin/orexin (Hcrt/Ox)-positive neurons^55^ in the LHA. We quantified the percentage of MCH neurons and Hcrt/Ox neurons that are cFos positive as a proxy for neuronal activity at three different states: at the beginning of the light phase (control baseline (B) group B), after 6 h of sleep deprivation (SD), and after 6 h of SD followed by a 4-h sleep rebound (RB; Fig. 4a,b) during which MCH neurons have been shown to be strongly active^19^. As expected, RB increased the percentage of cFos-positive (active) MCH neurons in *Wt* mice (Fig. 4c,e). In *App*^NL-G-F^ mice, however, this RB-induced increase was suppressed compared to *Wt* (Fig. 4c,e). The percentage of active Hcrt/Ox neurons did not differ between *Wt* and *App*^NL-G-F^ mice for any of the three states (Fig. 4d,e). Together, these results indicate a selective decrease in the percentage of active MCH neurons following RB sleep in *App*^NL-G-F^ mice at 6 months.

### Selective reduction in rapid eye movement sleep in *App*^NL-G-F^ mice

**Extended Data Fig. 7:**
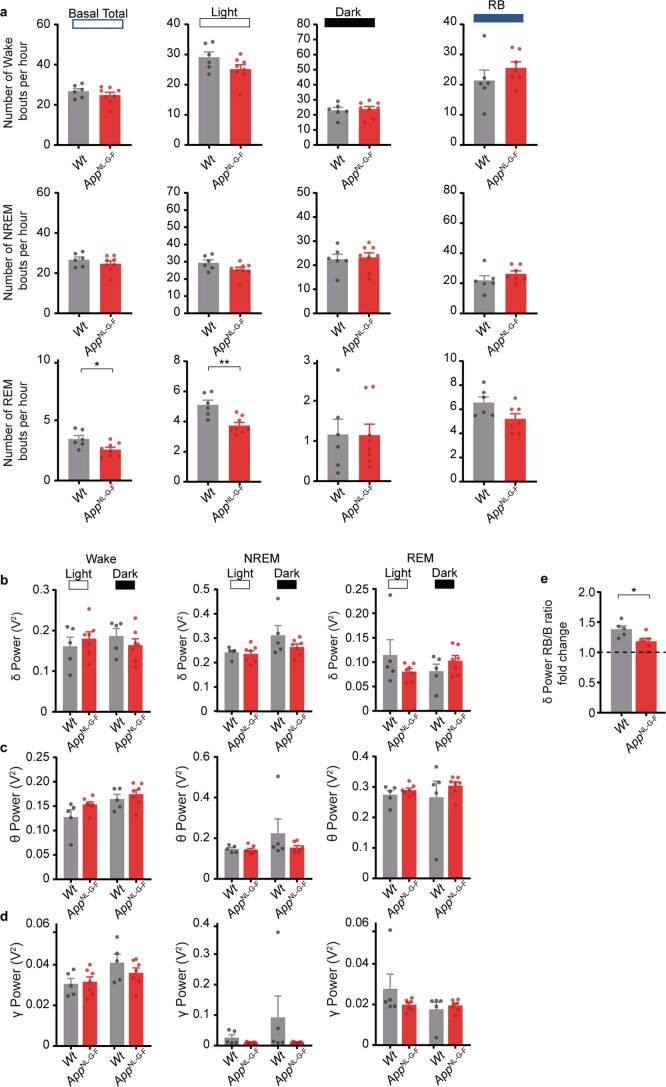
Sleep homeostatic response is impaired in *App*^NL-G-F^*mice*. **a**, EEG/EMG recordings showing the number of wake, NREM and REM bouts per hour during total basal recording, light or dark phase, and during RB sleep. Number of mice: 6 MO - B: *Wt* n = 6, *App*^NL-G-F^ n = 8; RB: *Wt* n = 6, *App*^NL-G-F^ n = 7 (REM: Basal Total p = 0.0225, Light p = 0.0022). One-way ANOVA with Tukey’s *post-hoc* test. Individual data points shown with bars representing mean ± SEM. (*p < 0.05, **p < 0.01). **b,c,d**, EEG/EMG recordings during basal recordings showing normalized power spectra of **(b)** delta, **(c)** theta and **(d)** gamma (30-60 Hz) bands in wake, NREM or REM states. Number of mice: 6 MO - B: *Wt* n = 5, *App*^NL-G-F^ n = 7. One-way ANOVA with Tukey’s post-hoc test or Kruskal-Wallis statistical test. Individual data points shown with bars representing mean ± SEM. **e**, Fold-change of normalized delta power spectrums during NREM sleep from EEG/EMG recordings during RB sleep. Number of mice: 6 MO - RB: *Wt* n = 5, *App*^NL-G-F^ n = 6 (p = 0.0277). Unpaired Mann-Whitney test. Individual data points shown with bars representing mean ± SEM. (*p < 0.05).

Active MCH neurons extend the duration of sleep, in particular REM sleep^18,56–58^. To determine whether sleep–wake architecture is altered in 6-month-old *App*^NL-G-F^ mice, we performed electroencephalographic (EEG) and electromyographic (EMG) recordings. Baseline EEG/EMG signal recordings showed no differences in time spent awake or in non-rapid eye movement (NREM) sleep between *Wt* and *App*^NL-G-F^ mice but revealed that *App*^NL-G-F^ mice spent less time in REM sleep during the light phase (Fig. 4f,g), in agreement with a previous study^59^. Additionally, *App*^NL-G-F^ mice showed a specific reduction in the number of REM sleep bouts per hour (Extended Data Fig. 7a). During baseline recordings, we detected no alterations on the cortical EEG oscillatory activities in *App*^NL-G-F^ mice compared to *Wt* mice (Extended Data Fig. 7b–d).

We then analyzed EEG/EMG signals during SD and RB sleep. Analysis of EEG/EMG signals during SD revealed that both genotypes were efficiently sleep deprived (*Wt* mice spent 96.6% of the time awake, 3.4% in NREM and 0% in REM sleep; *App*^NL-G-F^ mice spent 95.4% of the time awake, 4.6% in NREM and 0% in REM sleep). Quantification of sleep–wake cycle architecture during RB showed no significant difference between *Wt* and *App*^NL-G-F^ mice in the number of awake, NREM or REM bouts per hour (Extended Data Fig. 7a) or in time spent awake or in NREM sleep (Fig. 4h). However, the time spent in REM sleep was specifically reduced in *App*^NL-G-F^ mice compared to *Wt* mice (Fig. 4h). This reduction was associated with the decreased percentage of active MCH neurons in *App*^NL-G-F^ mice in the RB group (Fig. 4c,e). In addition, the sleep homeostatic response was impaired in *App*^NL-G-F^ mice during RB as these mice did not show an increase in NREM sleep delta power^60^ as observed in *Wt* mice (Extended Data Fig. 7e). Taken together, these results indicate a perturbation of the REM sleep component in *App*^NL-G-F^ mice at 6 months.

### Impaired melanin-concentrating hormone axon morphology in *App*^NL-G-F^ and individuals with Alzheimer’s disease

**Fig. 5:**
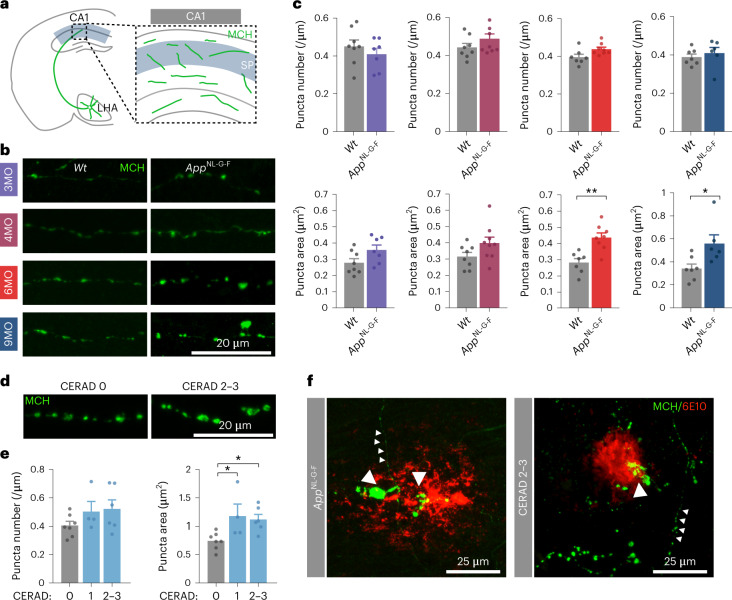
Progressive impairment in MCH axon morphology in *App*^NL-G-F^ mice and individuals with Alzheimer’s disease. **a**, Schematic showing location of analyzed axons. **b**,**c**, CA1-projecting MCH-positive axons in *Wt* and *App*^NL-G-F^ mice at 3, 4, 6 and 9 months. Representative images (**b**) and quantification (**c**) of the number of MCH puncta per axon length and respective puncta area. Number of mice: 3MO - *Wt* *n* = 8, *App*^NL-G-F^ *n* = 7, 4MO - *Wt* *n* = 8, *App*^NL-G-F^ *n* = 8, 6MO - *Wt* *n* = 7, *App*^NL-G-F^ *n* = 8 (*P* = 0.0014), 9MO - *Wt* *n* = 7, *App*^NL-G-F^ *n* = 6 (*P* = 0.023). Two-tailed unpaired *t*-test. Individual data points are shown; bars represent the mean ± s.e.m. (*P < 0.05, **P < 0.01). **d**,**e**, CA1-projecting MCH-positive axons in controls and individuals with AD at different stages of AD pathology. Representative images (**d**) and quantification (**e**) of the number of MCH puncta along axon length and respective puncta area in relation to CERAD. Number of individuals with AD: CERAD 0 *n* = 7; CERAD 1 *n* = 4 (*P* = 0.0404); CERAD 2–3 *n* = 6 (*P* = 0.0466). One-way ANOVA with Tukey’s post hoc test. Individual data points are shown; bars represent the mean ± s.e.m. (*P < 0.05). **f**, Representative images of MCH axons in CA1 hippocampal region show aberrant morphology near Aβ plaques labeled by 6E10 antibody (large arrowhead) and neurite with normal morphology (small arrowheads), in *App*^NL-G-F^ and brain samples from individuals with AD.

Our observations point to a decrease in active MCH neurons and dysregulation of sleep–wake architecture in 6-month-old *App*^NL-G-F^ mice. To determine whether we could detect evidence of an impairment of the MCH system at the hippocampal level, we analyzed morphological properties of MCH axons in *Wt* and *App*^NL-G-F^ mice in the CA1 region at different time points using immunohistochemistry for MCH. We found that the number of MCH-positive puncta per axon length was similar between the two genotypes at each time point, but that their area gradually increased in *App*^NL-G-F^ mice from 6 months onwards (Fig. 5a–c). This suggests that MCH peptide accumulates in axonal projections and fails to be released in *App*^NL-G-F^ mice at the stage when sEPSC frequency in CA1 pyramidal neurons starts to rise again (Fig. 1c).

**Extended Data Fig. 8:**
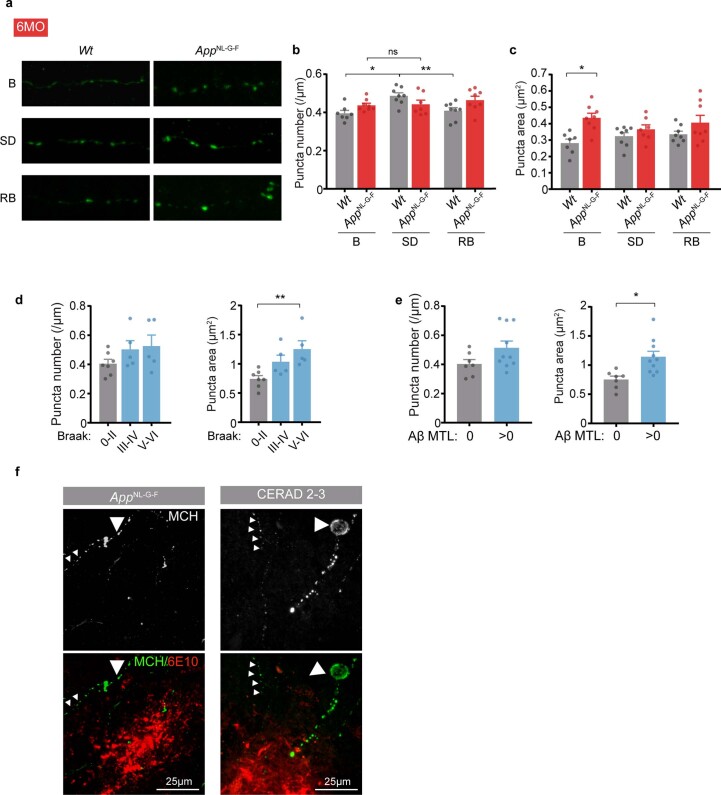
MCH-positive axon morphological changes. **a,b,c**, MCH-positive axons in CA1 region from *Wt* and *App*^NL-G-F^ mice at 6 months in B, SD and RB groups. **(a)** Representative image and **(b)** quantification of the number of MCH puncta along axon length (*Wt* B vs *Wt* SD p = 0.0079; *Wt* SD vs *Wt* RB p = 0.0266) and **(c)** respective puncta area (*Wt* B vs *App*^NL-G-F^ B p = 0.0093). Number of mice: B: *Wt* n = 7, *App*^NL-G-F^ n = 8; SD - *Wt* n = 8, *App*^NL-G-F^ n = 7, RB - *Wt* n = 8, *App*^NL-G-F^ n = 8. One-way ANOVA with Tukey’s *post-hoc* test. Individual data points shown with bars representing mean ± SEM. (*p < 0.05, **p < 0.01). Data shown for B group is the same as shown in Fig. 5c. **d, e**, Quantification of the number of MCH puncta per axon length and respective puncta area in relation to **(d)** Braak stages (p = 0.0083) and **(e)** Aβ deposition in the medial temporal lobe (Aβ MTL) (p = 0.006). Number of patients: Braak 0-II n = 7; Braak I-VI n = 5; Braak V-VI n = 5. One-way ANOVA with Tukey’s *post-hoc* test. Individual data points shown with bars representing mean ± SEM. (*p < 0.05). **f**, Representative images of MCH axons in CA1 hippocampal region showing aberrant morphology indicated by the large arrowhead near Aβ plaques labelled by 6E10 antibody and neurite with normal morphology indicated by smaller arrowheads, in *App*^NL-G-F^ and AD patient brain.

Axonal MCH peptide levels fluctuate with circadian phases and are higher during the dark phase^61^. To investigate whether peptide levels in MCH-containing axons in the CA1 region vary with sleep manipulation in *Wt* and *App*^NL-G-F^ mice, we analyzed MCH axons in CA1 following SD and RB sleep at 6 months. In *Wt* mice, the number of MCH puncta increased following SD and renormalized after RB sleep, with no significant changes in area size (Extended Data Fig. 8a–c). In contrast, neither SD nor RB affected the number of MCH puncta in *App*^NL-G-F^ mice (Extended Data Fig. 8a–c). These observations indicate a possible homeostatic regulation—such that in control conditions, MCH peptide levels in MCH axons increase with sleep pressure and decrease after RB sleep—that is compromised in 6-month-old *App*^NL-G-F^ mice and can lead to impaired trafficking or release of MCH peptide.

To determine whether these observations in *App*^NL-G-F^ mice are relevant for humans, we next analyzed MCH axon morphology in postmortem hippocampal sections from individuals with AD. As observed in *App*^NL-G-F^ mice, MCH axons in the CA1 region from individuals with AD with plaque deposition showed an increased area of MCH-positive puncta (Fig. 5d,e and Extended Data Fig. 8d–f). Importantly, this phenotype emerges with progression of the disease, as determined by the neuritic plaque score developed by the Consortium to Establish a Registry for Alzheimer’s Disease (CERAD; Fig. 5d,e), Braak neurofibrillary tangle staging and Aβ deposition in the medial temporal lobe (Extended Data Fig. 8d–f). MCH axons passing through Aβ plaques, revealed by 6E10 immunostaining, adopted a particularly aberrant morphology^62^ (Fig. 5f). Together, these observations show that MCH axon morphology in the CA1 region becomes progressively impaired in both *App*^NL-G-F^ mice and human AD brains.

## Discussion

Our findings indicate that homeostatic mechanisms transiently counteract but ultimately fail to stabilize, Aβ-induced aberrant excitatory drive of hippocampal CA1 neurons in *App*^NL-G-F^ mice^9^. Using spatial transcriptomics to obtain transcriptional profiles of CA1 pyramidal neuron cell bodies and of their surrounding cellular niches^26^, we identified changes in *Pmch* mRNA levels in CA1 as part of the adaptive response to increased excitatory synaptic transmission. This pointed to the involvement of the MCH system in AD pathophysiology.

MCH activity has been shown to modulate hippocampus-dependent functions^20,35,38,63,64^. The MCHR1 receptor is enriched in CA1 neuron cilia and MCH-neuron activity regulates cilia length^65^, supporting a role for MCH release in the CA1 region, but how MCH modulates hippocampal neuron activity remained largely unexplored. We find that MCH peptide downregulates spontaneous excitatory synaptic transmission and decreases MFR during spontaneous activity in hippocampal neurons, consistent with observations in other systems^66–68^. Moreover, MCH modulates the homeostatic response machinery, in part through a transcriptional response that involves suppression of genes regulating synaptic plasticity and neuronal excitability. These findings indicate that MCH modulates synaptic plasticity and excitability in hippocampal neurons. Consistent with this, we find that MCH peptide treatment reverses the aberrant excitatory drive observed in hippocampal slices derived from the *App*^NL-G-F^ AD mouse model.

Our findings identify the MCH system as vulnerable in early AD. MCH neurons are prominently active during REM sleep^54^. We find that a reduction in the percentage of active MCH neurons in *App*^NL-G-F^ mice is paralleled by a decrease in the time spent in REM sleep. Sleep is essential to transform awake experiences into consolidated memories and this process involves synaptic plasticity and remodeling processes that lead to a net decrease in neuronal firing rate, prominently during REM sleep^11–17,69^. Of note, decreased sleep quality is associated with AD pathology^8^ and reduced REM sleep correlates with the risk of dementia onset^70^.

Further pointing to an impaired function of the MCH system, we find that MCH axons projecting from the LHA to the hippocampal CA1 region show progressive alterations in *App*^NL-G-F^ mice and in individuals with AD, including enlarged MCH-containing boutons and the presence of large axonal swellings. Such axonal spheroids were recently demonstrated to disrupt long-range connectivity through a loss of electrical conduction along axons in an AD mouse model^71^. As a widely projecting system, MCH axons would be especially vulnerable to such plaque-induced pathology. The axonal conduction blockades could result in reduced peptide release, which generally requires strong stimulation, thereby impairing MCH function in the CA1 region.

**Extended Data Fig. 9:**
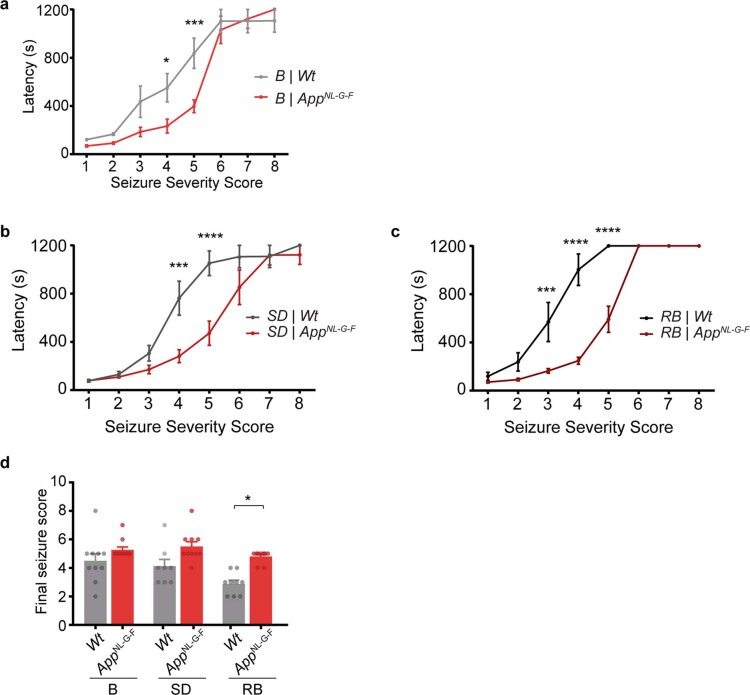
*App*^NL-G-F^ mice have an increased susceptibility to develop seizures. **a,b,c**, Time taken by *Wt* and *App*^NL-G-F^ mice to reach seizure level score (see method section) in response to i.p. injection of PTZ (40 mg/kg) at **(a)** B state (beginning of light phase) (stage 4: *Wt* vs *App*^NL-G-F^ p = 0.035, stage 5: *Wt* vs *App*^NL-G-F^ p = 0.0008) and **(b)** SD (stage 4: *Wt* vs *App*^NL-G-F^ p = 0.0003, stage 5: *Wt* vs *App*^NL-G-F^ p = 0.0001) and **(c)** after RB sleep (stage 3: *Wt* vs *App*^NL-G-F^ p = 0.0001, stage 4: *Wt* vs *App*^NL-G-F^ p = 0.0001, stage 5: *Wt* vs *App*^NL-G-F^ p = 0.0001). Number of mice: 6 MO - B: *Wt* n = 10, *App*^NL-G-F^ n = 11; SD: *Wt* n = 8, *App*^NL-G-F^ n = 10; RB: *Wt* n = 9, *App*^NL-G-F^ n = 10. Two-way ANOVA, with Holm-Sidak *post-hoc* multiple comparisons test. Individual data points shown with bars representing mean ± SEM. (*p < 0.05, **p < 0.01, ***p < 0.001). **d**, Mean score of seizure reached by *Wt* and *App*^NL-G-F^ mice in response to i.p. injection of PTZ (40 mg/kg) at B, SD and RB sleep. Number of mice: 6 MO - B: *Wt* n = 10, *App*^NL-G-F^ n = 11; SD: *Wt* n = 8, *App*^NL-G-F^ n = 10; RB: *Wt* n = 9, *App*^NL-G-F^ n = 10 (p = 0.0117). One-way ANOVA with Tukey’s *post-hoc* test. Individual data points shown with bars representing mean ± SEM. (*p < 0.05).

Our work suggests a model in which impaired MCH-dependent synaptic function in CA1 and perturbed REM sleep synergistically compromise neuronal homeostasis, contributing to aberrant neuronal activity in CA1, potentially increasing seizure susceptibility. We confirm that *App*^NL-G-F^ mice have an increased susceptibility to develop seizures upon an intraperitoneally injected subthreshold dose of the GABA_A_ receptor antagonist pentylenetetrazol (PTZ)^72^, and find that this is enhanced by acute sleep perturbations (Extended Data Fig. 9).

**Extended Data Fig. 10:**
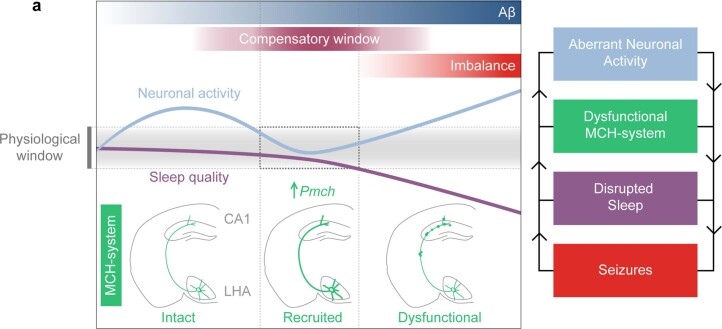
Working model. **a**, Schematic representation of the proposed working model.

The proposed model (Extended Data Fig. 10) provides a cellular and molecular basis for previously observed aberrant neuronal activity, sleep disturbances and impaired memory formation in the early stages of AD. Moreover, it supports the idea that failure of the cellular regulators of homeostasis in response to AD-related pathology plays a key role in the disease progression^73^. Consistent with our findings, intranasal infusion of MCH in an AD mouse model was recently shown to improve memory and reduce soluble Aβ levels^74^. However, the broad range of behavioral and metabolic effects resulting from manipulation of the MCH system^75^ warrants caution in directly translating these results into therapeutic options in humans. Finally, impairment of MCH-mediated neuronal homeostasis may be broadly relevant for other neurodegenerative^76^ and psychiatric disorders^77,78^.

## Methods

### Mice

All mouse lines were maintained on a C57BL/6J background, bred in-house and raised in a temperature-controlled and humidity-controlled room with a 14–10 h light–dark cycle (lights on from 7:00 to 21:00). *App*^NL-G-F^ knock-in^79^ mice express Swedish (p.LysMet670/671AsnLeu), Beyreuther/Iberian (p.Ile716Phe) and Arctic (p.Glu693Gly) mutations in the App gene under the endogenous promoter on the C57BL/6J background. *App*^NL-G-F^ mice were backcrossed for at least two generations with C57BL/6J mice. In the *Pmch*-cre, cre expression is driven by a ~108-kb fragment of the *Pmch* gene promoter using bacterial artificial chromosome technology^18^. Males and females are used (Supplementary Table 7). All experimental protocols were approved by the Institutional Animal Care and Research Advisory Committee of the KU Leuven (ECD P183/2017) and were performed in accordance with the Animal Welfare Committee guidelines of the KU Leuven, Belgium. The health and welfare of the animals was supervised by a designated veterinarian. The KU Leuven animal facilities complied with all appropriate standards (cages, space per animal, temperature, light, humidity, food and water), and all cages were enriched with materials that allow the animals to exert their natural behavior.

### Antibodies

Anti-mouse GluR1 (Milipore, MAB2263; 1:500 dilution), anti-rabbit MCH (H-070-034, Phoenix; 1:500 dilution), anti-rabbit GluR1 pSer845 (Millipore, AB5849; 1:500 dilution), anti-mouse 6E10 (803003, BioLegend; 1:1,000 dilution), anti-sheep Orexin (LS-B31, LSBio; 1:1,000 dilution), anti-mouse cFos (MCA-2H2, EnCore; 1:1,000 dilution), anti-rabbit cFos (Synaptic Systems, 226-003; 1:1,000 dilution), anti-rabbit Iba1 (234-003, Synaptic Systems; 1:1,000 dilution), anti-guinea pig GFAP (173-004, Synaptic Systems; 1:1,000 dilution).

### Patch-clamp electrophysiology in acute slices

Electrophysiology was performed at the VIB-KU Leuven Center for Brain and Disease Research Electrophysiology Expertise Unit. All recordings were performed on *Wt* or *App*^NL-G-F^ littermate pairs. For any parameter analyzed, a minimum of three pairs were used. Mice younger than 2 months were anesthetized with isoflurane and rapidly decapitated to prepare acute 300-μm-thick parasagittal brain slices on a Leica VT1200 vibratome. Mice older than 2 months were anesthetized with Nembutal and transcardially perfused with sucrose-based cutting solution (see below) to prepare acute 300-μm-thick parasagittal brain slices on a Leica VT1200 vibratome. Slicing was performed in a sucrose-based cutting solution (artificial cerebrospinal fluid, aCSF) that consisted of: 87 mM NaCl, 2.5 mM KCl, 1.25 mM NaH_2_PO_4_, 10 mM glucose, 25 mM NaHCO_3_, 0.5 mM CaCl_2_, 7 mM MgCl_2_, 75 mM sucrose, 1 mM kynurenic acid, 5 mM ascorbic acid and 3 mM pyruvic acid (pH 7.4 with 5% CO_2_/95% O_2_). Slices were allowed to recover at 34 °C for 30 min, and then maintained at room temperature in the same solution for at least 30 min before using. Pipettes were pulled on a horizontal micropipette puller (Sutter P-1000) and resistances ranged from 3 to 5 MΩ. Whole-cell voltage clamp recordings were made of CA1 pyramidal neurons at the distal region of pyramidal layer and data collected with pCLAMP 10. All recordings were done at 34 °C. Input resistance, pipette series resistance and membrane holding current were monitored throughout all recordings to ensure stability and quality. Currents were sampled at 20 kHz and stored after 3 kHz low-pass Bessel filtering (Molecular Devices DigiData 1440A and Multiclamp 700B). Before analysis, the data were low-pass filtered at 1 kHz.

For sEPSCs, slices were perfused at 1–2 ml min^−1^ with aCSF consisting of: 119 mM NaCl, 2.5 mM KCl, 1 mM NaH_2_PO_4_, 11 mM glucose, 26 mM NaHCO_3_, 4 mM MgCl_2_, 4 mM CaCl_2_ and 0.05 mM picrotoxin, bubbled continuously with 95% O_2_ and 5% CO_2_ using a cesium methanesulfonate-based internal solution: 115 mM cesium methanesulfonate, 20 mM cesium chloride, 10 mM HEPES, 2.5 mM MgCl_2_, 4 mM ATP disodium salt, 0.4 mM GTP sodium salt, 10 mM creatine phosphate and 0.6 mM EGTA, adjusted to a pH of 7.5 and 295 mOsm. Membrane potential was clamped at −70 mV. Then, 1 µM MCH peptide (H-070-47, Phoenix) was applied via perfusion into the bath recording solution after control baseline recording, and data were collected 10–15 min after perfusion started. sEPSCs were analyzed using the Mini Analysis program (Synaptosoft). Intrinsic properties were quantified using Clampfit 10.7 (Axon Instruments).

For intrinsic properties, we perfused at 1–2 ml min^−1^ with aCSF consisting of: 124 mM NaCl, 2.5 mM KCl, 1.2 mM NaH_2_PO_4_, 24 mM NaHCO_3_, 5 mM HEPES, 12.5 mM glucose, 2 mM MgSO_4_.7H_2_O, 2 mM CaCl_2_.2H_2_O and 0.05 mM picrotoxin bubbled continuously with 95% O_2_ and 5% CO_2_ and used a potassium gluconate-based internal solution: 135 mM potassium gluconate, 4 mM KCl, 2 mM NaCl, 10 mM HEPES, 4 mM EGTA, 4 mM Mg ATP and 0.3 mM Na GTP, adjusted to pH 7.25 and 295 mOsm. Somatic current injections of 20 pA steps starting at −50 until 530 pA were used for action potential profiling.

### Multielectrode array electrophysiology in acute slices

Whole-brain parasagittal sections were prepared as described above. Age-matched *Wt* and *App*^NL-G-F^ mice (approximately 6 months) were used for LTP experiments. For recordings, slices were placed onto a multielectrode array (MEA 2100, Multichannel Systems) and continuously perfused with 34 °C aCSF solution (119 mM NaCl, 2.5 mM KCl, 1 mM NaH_2_PO_4_, 11 mM glucose, 26 mM NaHCO_3_, 4 mM MgCl_2_ and 4 mM CaCl_2_) at pH 7.4, 95% O_2_ and 5% CO_2_. Field excitatory postsynaptic potentials (fEPSPs) were recorded from Schaffer collateral–CA1 synapses by stimulating and recording from the appropriate (visually identified) electrodes. Input–output curves were recorded for each slice by applying single stimuli ranging from 500 mV to 2,750 mV with 250-mV increments. Stimulus strength that corresponds to 35% of maximal response in the input–output curve was used for the following recordings. Paired-pulse facilitation experiments were performed by applying paired stimuli with 25, 50, 100, 200 and 400 ms inter-stimulus intervals. For LTP experiments, stable fEPSPs were recorded for 30 min to establish a baseline. For normal LTP induction, we applied three high-frequency trains (100 stimuli; 100 Hz) with 5-min intervals. For minimal LTP induction, we used two shorter trains (75 stimuli; 100 Hz) with a 5-min interval. Subsequently, post-LTP fEPSPs are measured every 5 min (average of three consecutive stimulations, 15 s apart) for 55 min. Recordings were analyzed and processed using Multi Channel Experimenter software (Multichannel Systems).

### Primary hippocampal neuronal cultures

Hippocampal neurons were cultured from embryonic day 18 C57BL/6 wild-type mice and 1 million neurons were plated on each well of a six-well plate coated with poly-d-lysine (Millipore) and laminin (Invitrogen). Neurons were maintained in Neurobasal Medium (Thermo Fisher Scientific, 21103049) supplemented with B27 (1:50 dilution; Thermo Fisher Scientific, 17504044), 12 mM glucose, glutamax (1:400 dilution; Thermo Fisher Scientific, 35050061), penicillin–streptomycin (1:500 dilution; Thermo Fisher Scientific, 15140122), 25 μM β-mercaptoethanol and 20 μg ml^−1^ insulin (Sigma-Aldrich, I9278). To prevent overgrowth of glia, neuron cultures were treated with 10 μM 5-fluoro-2′-deoxyuridine (Sigma-Aldrich, F0503) after 3 d. Neurons were treated with control vehicle (H_2_O) or 1 µM MCH peptide (H-070-47, Phoenix) for 4 h, and collected for immunoblotting or RNA extraction on day in vitro (DIV) 12–14.

### Patch-clamp electrophysiology in hippocampal neuronal cultures

Hippocampal neurons (DIV 11–16) were recorded after 4–6 h pretreatment with different concentrations of MCH peptide (100 nM, 300 nM and 1 µM) or equal volume of H_2_O as control vehicle. The intracellular whole-cell pipette medium contained: 136 mM KCl, 18 mM HEPES, 4 mM Na-ATP, 4.6 mM MgCl_2_, 15 mM creatine phosphate, 1 mM EGTA and 50 mM U ml^−1^ phospocreatine kinase (300 mOsm, pH 7.30). The regular external solution contained: 140 mM NaCl, 2.4 mM KCl, 2 mM CaCl_2_, 2 mM MgCl_2_, 10 mM HEPES, 14 mM glucose (300 mOsm, pH 7.30) and 20 µM bicuculline. Using whole-cell voltage clamp recording (double EPC-10 amplifier, Patchmaster v2x32 software, HEKA Elektronik; −70 mV), sEPSCs and mEPSCs in the presence of 1 µM tetrodotoxin (TTX) were recorded. Currents were recorded at 20 kHz and low-pass filtered at 3 kHz when stored. Patch pipettes (3 to 5 MΩ) were pulled from borosilicate glass using a multi-step puller (Sutter Instruments, P-1000). Series resistance was compensated to 70–75%. Only cells with series resistances < 15 MΩ were included in analysis. All recordings were done at room temperature. Spontaneous events were detected using Mini Analysis program (Synaptosoft).

### Multielectrode array electrophysiology in hippocampal neuronal cultures

Postnatal hippocampal cultures were plated on MEA plates containing 120 titanium nitride electrodes, in addition to 4 internal reference and 4 ground electrodes^42^. Each electrode has a diameter of 30 μm and electrodes were arranged in a 12 × 12 grid (sparing 6 electrodes in each corner), spaced 100–200 μm apart on average (Multi Channel Systems (MCS), 120MEA200/30iR-Ti). Data acquisition was done in 2-week-old cultures using a standard MEA2100-Systems and MEA2100-mini-Systems (MCS) with a hardware filter cutoff of 3.3 kHz and sampling rate of 10 kHz per electrode. Recordings were carried out under constant 37 °C and 5% CO_2_ levels.

### Spatial transcriptomics

The gene expression data obtained from spatial transcriptomics were derived from a dataset available in the host laboratory^26^. Raw data are available at GSE152506 (https://alzmap.org/). Briefly, 10-µm-thick coronal brain cryosections (bregma: −2.0 to −2.2) were obtained from 3.5- and 18-month-old *Wt* or *App*^NL-G-F^ mice, layered onto a spatially barcoded array of 1,007 TDs (diameter of 100 μm and a center-to-center distance of 200 μm, over an area of 6.2 mm by 6.6 mm) to collect in situ two-dimensional RNA-seq (10001, Spatial Transcriptomics). Each spot contains approximately 200 million barcoded reverse-transcription oligo(dT) primers allowing us to obtain a global transcriptomic profile of a TD with a volume of 0.00008 mm^3^ (pr2h with *r* = 50 μm and *h* = 10 μm). Images were acquired by a Zeiss Axio Scan.Z1 slide scanner (Carl Zeiss AG), and library preparation was performed after imaging following the Library Preparation Manual (Spatial Transcriptomics) as previously described^26^. We extracted expression data assigned to the hippocampal region of *Wt* and *App*^NL-G-F^ mice at 3.5 months of age for analysis. For comparative analysis, we also extracted expression data from the hippocampal region of mice at month 18, and from the hypothalamus (at month 3.5 and month 18).

#### Uniform manifold approximation and projection embeddings

We used Seurat (v3.1.4)^80^ to cluster hippocampal ST data as follows: normalized expression data (counts per million normalized to library size and log-transformed) was used as input. We scaled the expression data based on the 2,000 most variable genes as calculated using Seurat’s variance stabilizing transform algorithm, regressing on number of reads at the same time. A principal-component analysis was run, and we used the top ten principal components to calculate nearest neighbors and uniform manifold approximation and projection coordinates, leaving all other Seurat parameters as default.

#### Differential expression and enrichment

Differential expression of the CA1 pyramidal layer (sp)—comparing *Wt* or *App*^NL-G-F^ genotypes—was performed as described in the original publication. Briefly, generalized linear models were fit, and differential expression was tested using EdgeR’s quasi-likelihood *F* test^81^. We defined DE genes as those genes with FDR < 0.05 as significant. The full results of the DE analysis are available in Supplementary Table 1.

#### Gene Ontology

To test for enrichment of ST DE genes, we extracted the top 200 genes (according to *P* value) and submitted to Gorilla^82^, using default parameters. GO categories were sorted by *P* value and the top eight were ordered by normalized enrichment score (Fisher’s exact test). The five most enriched GO categories are shown in the bar plot. The genes annotated in each GO term are available in Supplementary Table 2.

#### Annotations in SynGO

We extracted the ST DE top 200 genes (according to *P* value) and submitted to the SynGO v1.1 database using default parameters (after using the SynGO conversion tool to convert mouse gene IDs to human IDs)^83^. SynGO genes are available in Supplementary Table 3.

#### Comparisons with homeostatic plasticity datasets

We extracted homeostatic synaptic plasticity data from the following studies: (1) RNA-seq data from a study of the transcriptional program responsible for synaptic upscaling during activity suppression (all genes affected by TTX or bicuculline^27^); (2) a characterization of the surfaceome of primary neuronal cultures (all total proteins and surface proteins changing with TTX or bicuculline treatment, *P* < 0.05; Supplementary Table 3);^28^ (3) a study of the synaptic proteome of the primary sensory cortex (proteins upregulated and downregulated in sensory-deprived cortex;^29^ (4) a single-cell RNA-seq study of activity-dependent transcriptional changes in mouse excitatory neurons^30^ (genes changing in clusters ExcL clusters in Supplementary Table 3). Protein names/IDs from proteomics data were converted to gene names using the UniProt mapping tool (https://www.uniprot.org/uploadlists/), or in the case where a match could not be found, manual annotation. This comparison is available in Supplementary Table 3.

#### Heat maps

Heatmaps were generated using log-normalized expression data (normalized using EgdeR’s cpm function), then scaled for each row. IDs from the original ST data have been modified for visualization purposes.

### Bulk RNA sequencing

For bulk RNA-seq, two independent cultures of hippocampal neurons were used. In each culture, we treated two wells with control vehicle (H_2_O) and two wells with 1 µM MCH peptide (H-070-47, Phoenix) for 4 h. RNA was extracted using the RNeasy Mini Kit (Qiagen 74104). RNA purity (260/280 and 260/230 ratios) and integrity were assessed using Nanodrop ND-1000 (Nanodrop Technologies) and an Agilent 2100 Bioanalyzer with High Sensitivity chips (Agilent Technologies) and a Qubit 3.0 Fluorometer (Life Technologies), respectively. RNA integrity values of the samples ranged from 7.9 to 9.3 (median, 8.6). Library preparation from total RNA extraction and sequencing were performed at the VIB Nucleomics Core. Briefly, 1 µg of total RNA extract per sample was enriched for mRNA molecules using poly-T oligonucleotide-attached magnetic beads. The enriched poly-A mRNA species was subjected to fragmentation and reverse transcription using random primers, and the subsequent library processing was done using the standard Illumina TruSeq Stranded mRNA Sample Prep Kit (protocol version 15031047 rev. E). Libraries were sequenced using an Illumina NovaSeq 6000 instrument at an average depth of approximately 31 million reads. Raw reads were pre-processed, mapped and quantified against the GRCm38 *Mus musculus* genome using the nf-core/rnaseq pipeline (v3.0; 10.5281/zenodo.1400710).

#### Differential analysis

Salmon quantifications from the nf-core/rnaseq pipeline were imported into R v4.0.3 using the tximport library. Resulting counts were normalized and differential analysis was performed using DEseq2 (v.1.13.0). Genes were tested for differential expression between vehicle-treated and MCH-treated samples and those with an adjusted *P* value (Benjamini–Hochberg) < 0.05 were deemed significant.

#### Functional analysis

To test for enrichment of DE genes, the top 200 genes (according to *P* value) were submitted to Gorilla^82^ to test for enrichment, using default parameters.

The data generated are available in the Gene Expression Omnibus (GEO; GSE225181)

### Immunohistochemistry, imaging and quantification

Mice were anesthetized with ketamine (0.2 mg per gram body weight) and xylazine (0.02 mg per gram body weight) intraperitoneally administered, perfused with 1× PBS for 1 min followed by 10 min of 4% paraformaldehyde (PFA) in PBS. Brains were post-fixed for 4 h in 4% PFA, washed in 1× PBS and embedded in 3% agarose. Then, 50-µm sections were prepared using a vibratome (Campden Instruments 7000smz). Sections were permeabilized with 0.5% Triton in PBS-0.2% gelatin for 30 min, blocked for 2 h in 10% normal horse serum and 0.5% Triton in PBS-0.2% gelatin. Primary antibodies were incubated for 48 h and secondary antibodies for 24 h. Primary and secondary antibodies were diluted in 5% normal horse serum and 0.5% triton in PBS-0.2% gelatin. Hoechst was used as a nuclear stain (5 nM in PBS).

#### cFos CA1 region

Confocal images were taken on a Leica TCS SP8 at ×63 magnification. Analysis was performed with IMARIS 9.5.1. Four to six fields of CA1 were imaged within the anteroposterior coordinates of approximately −1.5 mm to −2.0 mm from bregma. A region of interest (ROI) delineating CA1 pyramidal layer was drawn. Next, the total number of cells was counted based on Hoechst signal using the surface tool to create a three-dimensional object. The percentage of cFos-positive cells was taken based on the number of Hoechst objects that are positive for cFos. For cFos intensity, mean values of intensity were taken for each cFos-positive nuclei surface. For normalization, an average of the values obtained for the *Wt* condition was calculated and each individual value was normalized to the *Wt* average. Analysis was done blind with regard to genotype and time point. Tissues from both genotypes of each time point were collected simultaneously, at the beginning of the light phase.

#### cFos lateral hypothalamic area region

Confocal images were taken on a Leica TCS SP8 at ×20 magnification. Analysis was performed with IMARIS 9.5.1. Four to six fields of the LHA, containing a minimum of five MCH-positive and five Hcrt/Ox-positive neurons were imaged within the anteroposterior coordinates of approximately −1.00 mm to −2.5 mm from bregma. The total number of MCH-positive or Hcrt/Ox-positive cells was counted manually. cFos-positive cells were visualized using the spot tool, and the percentage of MCH or Hcrt/Ox cells positive for cFos was manually determined by counting how many MCH-positive or Hcrt/Ox-positive cells overlapped with the cFos signal. Analysis was done blind with regard to genotype and sleep manipulation. Tissues from both genotypes of each time point were collected simultaneously, at the beginning of the light phase for basal (B) group or according to the sleep manipulation protocol.

#### Spine analysis

For spine analysis, 80-µm sections were prepared and immunostained with anti-GFP, and CA1 pyramidal neurons were imaged with a Zeiss LSM880 confocal microscope with an Airyscan detector. Around 3–4 neurons were selected per animal and 2–3 secondary dendrites were randomly selected within this ROI for analysis. Spines were quantified only from dendrites with a length of at least 20 μm. Dendritic protrusions and length were quantified in Imaris software. Analysis was done blind with regard to genotype and time point. Tissues from both genotypes of each time point were collected simultaneously, at the beginning of the light phase.

#### Melanin-concentrating hormone morphology analysis

MCH-positive axons in all laminas of the CA1 region were imaged on an LSM880, at a magnification of ×63 and zoom of ×1. Stacks of 1 μm in step size were acquired. Analysis was performed using ImageJ. Axon length was determined. MCH-positive puncta were determined using the threshold tool, and number and areas of puncta were determined using the Analyze Particles tool. Particle numbers were normalized to axon length. Analysis was done blind with regard to genotype, sleep manipulation and time point. Tissues from both genotypes of each time point were collected simultaneously, at the beginning of the light phase for the basal (B) group or according to the sleep manipulation protocol.

#### Sleep manipulation

For the basal (B) group: animals were perfused at the beginning of the light phase; for group SD: animals underwent SD from the beginning of the light cycle for 6 h and were perfused; group RB sleep (RB): animals underwent SD from the beginning of the light cycle for 6 h, and were allowed to RB sleep for 4 h, before perfusion. SD started at the beginning of light phase. Animals were kept awake by gentle touch with a brush. At the 3rd and 4th hour of SD, a novel object was inserted in each cage. Gentle poking with a brush continued until 6 h of SD.

### Human tissue

Brain tissues were collected in accordance with the applicable laws in Belgium and Germany. The recruitment protocols for collecting the brains received from the Municipal hospital in Offenbach/Main (Germany) were approved by the ethical committees of the University of Ulm (Germany) and of UZ Leuven (Belgium). This study was approved by the UZ Leuven ethical committee (Belgium). Brains were fixed in a 4% aqueous solution of formaldehyde for approximately 2–4 weeks. The brain hemispheres were cut into 1-cm frontal slabs and stored in polyethylene glycol. Medial temporal lobes were dissected and embedded partially in polyethylene glycol and partially in paraffin. For neuropathological diagnosis, paraffin sections were stained with hematoxylin and eosin, the Gallyas silver method, p-tau (AT8, Pierce; 1:1,000 dilution) and Aβ (4G8, Senetec; 1:5,000 dilution; formic acid pretreatment). The Braak neurofibrillary tangle stages^84^ and the phase of Aβ plaque deposition^85^ were determined as recommended to determine the degree of AD pathology^86^. None of the cases included in this study showed signs of hypoxemia-related neuron damage. For immunohistochemistry, 150–200 µm PEG sections were prepared with a vibratome and transferred to 70% ethanol solution. Briefly, sections were incubated with 88% formic acid and 0.1% sodium borohydride for 20 min. Immunohistochemistry in human sections was performed as described above for mouse sections. Primary and secondary antibodies were incubated for 48 h.

Ethical approval by the UZ Leuven ethical committee (Leuven/Belgium; decision no. S-S63259). An informed consent for autopsy and scientific use of autopsy tissue with clinical information was granted according to local legislation. All methods have been performed in accordance with the relevant guidelines and regulations.

### Adeno-associated virus

Adeno-associated virus (AAV) 9 hSyn-DIO-mCherry was acquired from Addgene (50459). Plasmids pCAG-GFP plasmid (11150, addgene) and pAAV-EF1a-DIO-hM3D(Gq)-mCherry (50460, Addgene) were used to produce AAVs in-house as briefly described: HEK293T cells were seeded in DMEM (Invitrogen) containing 10% FBS (Invitrogen). Transfection mix, containing PEI and OptiMEM (Invitrogen) and adenovirus helper plasmid (pAdΔF6), a packaging plasmid pAAV2/1 rep-cap 2,1 (Pennvector Core PL-T-PV0001) and vector plasmid of interest, were added to the cells in DMEM containing 1% FBS. Cells were incubated in DMEM containing 5% FBS for 3 d. Cells were harvested, centrifuged at 1,000*g* at 4 °C for 10 min and pellets were lysed in lysis buffer (150 mM NaCl and 50 mM Tris HCl pH 8.5). Lysates were further frozen and thawed three times, centrifuged at 2,000*g* at 4 °C for 5 min and Benzonase (Sigma) added at a concentration of 50 U ml^−1^ to supernatants for 30 min at 37 °C. Lysates were centrifuged at 5,000*g* for 20 min at room temperature. OptiPrep iodixanol (Sigma) gradients of 15%, 25%, 40% and 60% were prepared with 5 M NaCl, 5× PBS with 1 mM MgCl_2_ and 2.5 mM KCl (5× PBS-MK) and sterile H_2_O, and layered in 25 × 77 mm OptiSeal tubes (Beckman Coulter). Supernatants loaded on top of gradients and centrifuged at 300,000*g* and 12 °C for 100 min in the Optima XE-100 Ultracentrifuge (Beckman Coulter). Next, AAVs were collected with an 18-gauge needle (Beckman Coulter) from between the 40% and 60% layers, and diluted in 5 ml 1× PBS-MK. AAVs were desalted and concentrated by centrifugation at 5,000*g* for 30 min at 20 °C in a prerinsed Amicon Ultra-15 filter (Millipore) in 1× PBS-MK, aliquoted and stored at −80 °C. Purity was assessed by SDS–PAGE and silver staining.

### Stereotaxic injection

Mice were intraperitoneally injected with buprenorphine at 0.05 mg per kg body weight (Vetergesic) and anesthetized with 5% isoflurane. Duratears was applied to the eyes. Mice were placed in a mouse stereotaxic frame equipped with gas anesthesia head holder (KOPF). During the rest of the procedure, 2.5% isoflurane was constantly administered. After shaving and disinfecting the mouse’s head, local anesthesia was administered by a subcutaneous injection with 100 µl lidocaine (xylocain 1%). After 5 min, an incision was made in the skin. AAVs were injected using a glass pipette pulled on a Sutter P-1000 and placed on a Nanoject III (Drummond) for loading control. The pipette was slowly lowered to the target site and remained in place for 1 min. Next, 50 nl of virus was injected at 1 nl s^−1^ and the pipette was removed 5 min after infusion was complete. After capillary removal, the burr hole was filled with bone wax (Fine Science Tools, 19009-00). The skin was closed using veterinary tissue adhesive (Dermafuse). Post-surgery analgesia buprenorphine (0.1 mg per kg body weight) was administered 4–6 h after surgery.

CA1 region (for spine analysis): anteroposterior: −2.4 mm from bregma, mediolateral: ±2.0 mm from sagittal suture, −1.5 mm dorsoventral relative to surface of the skull. Mice were kept for 30 d before perfusion and spine analysis.

LHA region (for RT–qPCR): −0.9 mm from bregma, mediolateral: ±0.5 mm from sagittal suture, −5.35 mm dorsoventral relative to surface of the skull. Mice were kept for 15 d before RNA extraction.

### RNAscope

Brains were freshly dissected and frozen in OCT compound and isopentane. Sections (10 µm) were prepared on a cryostat (Leica) and fixed in 4% PFA for 10 min. RNAscope hybridization was performed using the RNAscope Multiplex Fluorescent Reagent Kit v2 Assay (Advanced Cell Diagnostics). After 4 × 10 min PBS washes, tissue sections were treated with pretreatment solutions and then incubated with RNAscope probes (*Mchr1*, *Pmch*, *vGlut2 (Slc17a6)*, *Gad2*, *Vglut1 (Slc17a7)*, *Itgam* and *Adhl1l*) followed by amplifying hybridization processes. DAPI was used as a nuclear stain. Prolong Gold Antifade (Thermo Scientific) was used to mount slides. Confocal images were taken on a Leica TCS SP8 microscope. Tile scans were taken on Axio Scan.Z1 slide scanner (Zeiss).

### RT–qPCR and DREADDs injection

The LHA of *Pmch*-cre mice was stereotaxically injected with 150–200 nl of AAV to express mCherry or hM3D(Gq)-mCherry (Supplementary Table 7). CNO dihydrochloride (HB6149, Hello Bio) was injected intraperitoneally at the beginning of the light phase for 4 h (3 mg per kg body weight). Next, hippocampi were dissected in ice-cold HBSS (14175095, Fisher Scientific) and snap frozen until further processing. Hippocampi were homogenized in RLT buffer (74106, Qiagen RNeasy Mini Kit) supplemented with 1% β-mercaptoethanol and using a 1-ml dounce tissue grinder. RNA was extracted from hippocampal homogenates using an RNeasy Mini Kit (74106, Qiagen) and following the manufacturer’s instructions, including DNase treatment (79256, Qiagen). Next, RNA was retrotranscribed to cDNA using High-Capacity cDNA Reverse Transcription Kit (4368814, Thermo Fisher) and following manufacturer’s instructions. Each sample was quantified by means of a quantitative PCR (qPCR) assay following manufacturer’s instructions (4707516001, LightCycler 480 SYBR Green I Master cDNA) and using Roche’s Real time PCR quantification LC480 instrument. For each qPCR reaction, 10 ng cDNA was used and three technical replicates per condition and gene, with specific primers listed below.

**Table:** 

Gene	Primer sequences	Notes
*PGK1* - forward	ACTGTGGCCTCTGGTATACCTG	Housekeeping- designed with Benchling
*PGK1* - reverse	CAATCTGCTTAGCTCGACCCAC	Housekeeping- designed with Benchling
*Pmch* - forward	GTCTGGCTGTAAAACCTTACCTC	Ref. ^87^
*Pmch* - reverse	CCTGAGCATGTCAAAATCTCTCC	Ref. ^87^

### Western blot

Laemmli buffer (4×; 8% SDS, 40% glycerol, 20% β-mercaptoethanol, 0.01% bromophenol blue and 250 mM Tris HCl pH 6.8), pH-adjusted with 1.5 M Tris HCl pH 8.8, was added to primary cultures at a 1× final concentration. Samples were boiled at 95 °C for 5 min, and loaded in a 4–12% polyacrylamide gels (Invitrogen). Protein was transferred into a nitrocellulose membrane using the semi-dry Trans-Blot Turbo Transfer System (Bio-Rad, 1704150). Total protein was quantified using the REVERT Total Protein Stain Kit (Licor LI 926-11010). A 5% milk solution in TBS-T (150 mM NaCl, 20 mM Tris, 0.5% Tween) was used to block and to prepare primary and secondary antibodies. SuperSignal West Femto Maximum Sensitivity Substrate (Thermo Scientific, 1859290) was used to develop western blots. ImageJ was used to quantify intensities within ROIs. For total protein quantification, intensity was averaged across three ROIs per lane, and ROI masks were placed at the same position for all lanes.

### ELISA detection of soluble and insoluble Aβ_42_

Hippocampi from *App*^NL^ or *App*^NL-G-F^ mice at 1, 2, 3 and 6 months were dissected after transcardial perfusion with ice-cold PBS. Tissue was homogenized in protein extraction reagent (Pierce). Homogenates were centrifuged at 4 °C for 1 h at 100,000*g* (Beckman TLA 100.4 rotor) and supernatants used for ELISA. Guanidine-HCl extraction protocol was used to extract GuHCl-soluble Aβ fraction. Aβ_42_ levels were quantified on Meso Scale Discovery (MSD) 96-well plates by ELISA using end-specific antibody provided by M. Mercken (Janssen Pharmaceutica, Belgium). Monoclonal antibody JRFcAβ42/26 against the C terminus of Aβ42 species was used as capture antibody and JRF/AβN/25 labeled with sulfo-TAG was used as the detection antibody. The plate was read in MSD Sector Imager 6000.

### EEG/EMG surgery

Animals were chronically implanted with EEG/EMG electrodes on a stereotaxic frame under isoflurane inhalation anesthesia as previously described^18^. Stainless-steel screws for EEG recordings were inserted into the skull (1) above the frontal cortex, (2) above the parietal cortex, and (3) a ground electrode was placed in midline above the cerebellum. All cortical electrodes were fixed to the skull via Tetric EvoFlow cement by Ivoclar vivadent. EMG signals were recorded from two pairs of electrodes sutured to the trapezoid muscles. Following surgery, animals were housed individually in their recording cage for 2–3 d for recovery from surgery, and their electrodes were connected to a digitizing head stage (RHD2132, Intan Technologies) and recording cable for habituation for a period of 10 d. Light–dark cycles were 12 h each with light phase starting at 8:00.

#### Sleep manipulation

For the basal group (B), EEG/EMG recordings started at the beginning of the light phase for 24 h; for group SD, animals underwent SD for 4 h; for group RB sleep, animals were allowed to RB sleep for 4 h. SD started at the beginning of the light phase. Animals were kept awake by gentle touching with a brush. At the 3rd hour of SD, a novel object was inserted in each cage. At and 4th hour of SD, animals were placed in a novel cage and returned to the recording cage before RB recording.

### Polysomnographic recording and data acquisition

Twenty-four-hour basal (B) recordings were performed starting at 8:00, followed by 4-h recordings during SD and a 4-h recording during RB sleep. EEG and EMG signals from electrodes were amplified (Grass Instruments), digitized at a sampling rate of 8 kHz and downsampled at 1 kHz, collected on a PC within the recording room using open-source software from Intan Technologies (RHD2000).

The polysomnographic recordings were visually scored offline using a custom software written in MATLAB, as either wake, NREM sleep or REM sleep. Briefly:

- Wakefulness—periods with low-amplitude desynchronized EEG signals and high, tonic EMG activity containing phasic bursts.
- NREM sleep—characterized by synchronized, high-amplitude oscillations in the slow wave and delta band and low EMG tone without bursts.
- REM sleep was scored when the EEG recording showed pronounced theta oscillations with almost complete absence of EMG tone except short twitches.

To generate power spectra during wakefulness, NREM and REM across the light and dark period, scored EEG signals were extracted using Welch’s method. Power spectra were calculated from 0.5 Hz to 500 Hz with a 0.5-Hz bin width. Power values were normalized to the sum of all power values from 0.125 Hz to 100 Hz. Power values were then calculated for the following frequency bands: delta (0.5–4.5 Hz), theta (6–10 Hz) and low gamma (20–60 Hz).

### Seizure threshold

For the seizure threshold score, mice were injected with a subthreshold dose of PTZ (40 mg per kg body weight, intraperitoneally) and placed individually in a recording chamber. Video recording started immediately after PTZ injection. Seizure severity score classification was performed upon video assessment as previously described^21^ as: 0 = normal behavior; 1 = immobility; 2 = generalized spasm, tremble or twitch; 3 = tail extension; 4 = forelimb clonus; 5 = generalized clonic activity; 6 = bouncing or running seizures; 7 = full tonic extension; 8 = death; with latency time recorded in a double-blind manner. For the basal group (B), PTZ was administered at the beginning of the light phase. For the sleep-deprived group (SD), PTZ was administered at the end of the manipulation.

#### Sleep manipulation

For the basal group, animals were injected at the beginning of the light phase; for group SD, animals underwent SD from the beginning of light cycle for 6 h and were injected; for group RB, animals underwent SD from the beginning of the light cycle for 6 h, were allowed to RB sleep for 4 h, before injection. SD started at the beginning of light phase. Animals were kept awake by gentle touching with a brush. At the 3^rd^ and 4^th^ hour of SD, a novel object was inserted in each cage. Gentle poking with a brush continued until 6 h of SD.

### Statistical analysis

Data were plotted in GraphPad Prism 8. For quantification, datasets were tested for normality using Shapiro–Wilk normality test. If datasets passed the test, they were analyzed using Student’s unpaired *t*-test. Otherwise, the datasets were analyzed using nonparametric unpaired *t*-test/Mann–Whitney test. One-way ANOVA test was used for multiple comparisons. Two-way ANOVA was used to compare datasets across multiple factors. Sample analysis was randomized. No statistical methods were used to predetermine sample sizes, but our sample sizes are similar to those reported in previous publications^59,88,89^. No data or animals were excluded from the study.

### Reporting summary

Further information on research design is available in the Nature Portfolio Reporting Summary linked to this article.

## Online content

Any methods, additional references, Nature Portfolio reporting summaries, source data, extended data, supplementary information, acknowledgements, peer review information; details of author contributions and competing interests; and statements of data and code availability are available at 10.1038/s41593-023-01325-4.

## Supplementary information

Reporting Summary

Supplementary Tables 1–7

## Data Availability

The data generated in this study are available in the GEO database (GSE225181). Spatial transcriptomics datasets from Chen et al.^26^ are available via the GEO under accession number GSE152506 (https://alzmap.org/). No new code or algorithms were generated for this publication. Source data are provided with this paper.

## Source data

Source Data Fig. 3 Unprocessed western blots.
