# Supplementary material for: Early alterations in the MCH system link aberrant neuronal activity and sleep disturbances in a mouse model of Alzheimer’s disease
Source: Nat Neurosci. 2023 May 15;26(6):1021–31. doi: 10.1038/s41593-023-01325-4 (PMC10244178; doi:10.1038/s41593-023-01325-4)
Supplement: Supplementary file 1 — Reporting Summary [file 41593_2023_1325_MOESM1_ESM.pdf]

nature portfolio

Corresponding author(s): Calafate, De Strooper, De Wit

Last updated by author(s): Apr 7, 2023

Reporting Summary

Nature Portfolio wishes to improve the reproducibility of the work that we publish. This form provides structure for consistency and transparency in reporting. For further information on Nature Portfolio policies, see our Editorial Policies and the Editorial Policy Checklist.

Statistics

For all statistical analyses, confirm that the following items are present in the figure legend, table legend, main text, or Methods section.

n/a	Confirmed	
☐	☒	The exact sample size (*n*) for each experimental group/condition, given as a discrete number and unit of measurement
☐	☒	A statement on whether measurements were taken from distinct samples or whether the same sample was measured repeatedly
☐	☒	The statistical test(s) used AND whether they are one- or two-sided<br>*Only common tests should be described solely by name; describe more complex techniques in the Methods section.*
☐	☒	A description of all covariates tested
☐	☒	A description of any assumptions or corrections, such as tests of normality and adjustment for multiple comparisons
☐	☒	A full description of the statistical parameters including central tendency (e.g. means) or other basic estimates (e.g. regression coefficient) AND variation (e.g. standard deviation) or associated estimates of uncertainty (e.g. confidence intervals)
☐	☒	For null hypothesis testing, the test statistic (e.g. *F*, *t*, *r*) with confidence intervals, effect sizes, degrees of freedom and *P* value noted<br>*Give P values as exact values whenever suitable.*
☒	☐	For Bayesian analysis, information on the choice of priors and Markov chain Monte Carlo settings
☒	☐	For hierarchical and complex designs, identification of the appropriate level for tests and full reporting of outcomes
☒	☐	Estimates of effect sizes (e.g. Cohen's *d*, Pearson's *r*), indicating how they were calculated

Our web collection on statistics for biologists contains articles on many of the points above.

Software and code

Policy information about availability of computer code

Data collection	PatchClamp in slices: Molecular Devices DigiData 1440A and Multiclamp 700B<br>PatchClamp in primary cultures: double EPC-10 amplifier, Patchmaster v2x32 software (HEKA Elektronik<br>Spatial Transcriptomics: Data available on GEO accession number GSE152506 (Chen et al., 2020)<br>Microscopy imaging: Leica TCS SP8 and Zeiss LSM880 confocal microscope with an Airyscan detector.<br>MEAs for slices: MEA 2100, Multichannel Systems<br>MEAs for primary cultures: MEA2100-Systems and MEA2100-mini-Systems<br>Bulk RNAsequencing: Library preparation with Illumina NovaSeq6000<br>EEG/EMG: EEG and EMG signals from electrodes were amplified (Grass Instruments, USA), digitized at a sampling rate of 8 kHz and downsampled at 1kHz, collected on a PC within the recording room using open-source software from Intan Technologies (RHD2000)

Data analysis	PatchClamp data: sEPSCs were analysed using the Mini Analysis program (Synaptosoft). Intrinsic properties were quantified using Clampfit 10.7 (Axon Instruments).<br>Spatial Transcriptomics: UMAP embeddings. We used Seurat v3.1.4 to cluster hippocampal ST data,<br>Microscopy imaging: ImageJ and Imaris 9.5.1. 4<br>MEAs for slices: Multi Channel Experimenter software (Multichannel Systems)<br>Bulk RNAsequencing: Raw fastq data was processed into gene counts using the nfcore/rnaseq nextflow pipeline (v3.0) with the GRCm38 genome option, the resulting counts from salmon were loaded into R (v4.0.3) using tximport (v1.18.0) and differential analyses were carried out using DEseq2 (v1.30.0).<br>EEG/EMG: The polysomnographic recordings were visually scored offline using a custom software written in Matlab. Analysis pipeline available in Facchin et al 2020, Oesch et al 2020 and Aime et al 2022

For manuscripts utilizing custom algorithms or software that are central to the research but not yet described in published literature, software must be made available to editors and reviewers. We strongly encourage code deposition in a community repository (e.g. GitHub). See the Nature Portfolio guidelines for submitting code & software for further information.

Data

Policy information about availability of data

All manuscripts must include a data availability statement. This statement should provide the following information, where applicable:

- Accession codes, unique identifiers, or web links for publicly available datasets
- A description of any restrictions on data availability
- For clinical datasets or third party data, please ensure that the statement adheres to our policy

The BulkRNAsequencing data generated in this study is available in Gene Expression Omnibus (GEO; GSE225181).
Spatial Transcriptomics datasets from Chen et al. are available via the GEO database (GEO accession number GSE152506).

Human research participants

Policy information about studies involving human research participants and Sex and Gender in Research.

Reporting on sex and gender	Brains from males and females were included
Population characteristics	Postmortem tissue available in the lab of Prof. Dietmar Thal were used. Only hippocampi were used. Brain diagnosis ranged from normal brain, with infarcts, with carcinoma, argyrophilic grain disease, MCI and AD. Additional information (e.g. age) is provided in Supplemental Table 6.
Recruitment	postmortem tissue used
Ethics oversight	Ethical approval by the UZ Leuven ethical committee (Leuven/Belgium; Decision-No. S-S63259)

Note that full information on the approval of the study protocol must also be provided in the manuscript.

Field-specific reporting

Please select the one below that is the best fit for your research. If you are not sure, read the appropriate sections before making your selection.

☒ Life sciences ☐ Behavioural & social sciences ☐ Ecological, evolutionary & environmental sciences

For a reference copy of the document with all sections, see nature.com/documents/nr-reporting-summary-flat.pdf

Life sciences study design

All studies must disclose on these points even when the disclosure is negative.

Sample size	Sample size was determined based on previous experiments in the lab.
Data exclusions	No data exclusions
Replication	For all experiments, a minimum number of animals used was applicable. Littermates were used whenever possible. For image analysis, a minimum number of images was taken per mouse. For electrophysiology recordings, a minimum number of neurons was recorded per mouse. Time of the day for analysis or samples collection was the same for all samples of the same experimental groups in order to decrease variability.
Randomization	Mice were randomly assigned to groups while ensuring an even distribution of sexes.
Blinding	Experimenters were blinded to conditions for quantitative imaging experiments.

Reporting for specific materials, systems and methods

We require information from authors about some types of materials, experimental systems and methods used in many studies. Here, indicate whether each material, system or method listed is relevant to your study. If you are not sure if a list item applies to your research, read the appropriate section before selecting a response.

Materials & experimental systems

n/a	Involved in the study
☐	☒ Antibodies
☒	☐ Eukaryotic cell lines
☒	☐ Palaeontology and archaeology
☐	☒ Animals and other organisms
☒	☐ Clinical data
☒	☐ Dual use research of concern

Methods

n/a	Involved in the study
☒	☐ ChIP-seq
☒	☐ Flow cytometry
☒	☐ MRI-based neuroimaging

Antibodies

Antibodies used

Anti-mouse GluR1 (Milipore MAB2263, 1/500)
anti-rabbit MCH (H-070-034, Phoenix, 1/500)
anti-rabbit GluR1 pSer845 (Millipore AB5849, 1/500)
anti-mouse 6E10 (803003, Biolegend 1/1000)
anti-sheep Orexin (LS-B31, LSBio, 1/1000)
anti-mouse cFos (MCA-2H2, EnCore, 1/1000)
anti-rabbit cFos (Synaptic Systems 226-003, 1/1000)
anti-rabbit Iba1 (234-003, Synaptic Systems, 1/1000)
anti-guinea pig GFAP (173-004, Synaptic Systems, 1/1000)

Validation

All antibodies are from commercially available sources and have been validated by the manufacturer with supporting publications found on manufacturer websites.

Animals and other research organisms

Policy information about studies involving animals; ARRIVE guidelines recommended for reporting animal research, and Sex and Gender in Research

Laboratory animals

All mouse lines were maintained on a C57BL/6J background, bred in-house and raised in a temperature- and humidity-controlled room with a 14-10h light–dark cycle (lights on from 7h00 to 21h00). AppNL-G-F mice: Apptm3.1Tcs/Apptm3.1Tcs (Saito et al, 2014); Pmch.cre: C57BL/6-Tg(Pmch-cre)1Rck/J (Jego, 2013). Additional information (e.g. age of mice used) is providd in Suppleementary Table 7.

Wild animals

n/a

Reporting on sex

Animals from diffferent sex were used in all experimental groups, with the exception of EEG/EMG recordings were only males were used due to technical requirements.

Field-collected samples

n/a

Ethics oversight

Animal experiments were approved by the local Ethical Committee of Laboratory Animals of the KU Leuven

Note that full information on the approval of the study protocol must also be provided in the manuscript.
